# Clovis organizational dynamics at a Late Glacial campsite in the central Great Lakes: Belson site excavations 2020–2021

**DOI:** 10.1371/journal.pone.0302255

**Published:** 2024-05-29

**Authors:** Brendan Nash, Thomas Talbot, Henry T. Wright, Elliot Greiner, Linda Scott Cummings

**Affiliations:** 1 Great Lakes Range, Museum of Anthropological Archaeology, University of Michigan, Ann Arbor, Michigan, United States of America; 2 Museum of Anthropological Archaeology, University of Michigan, Ann Arbor, Michigan, United States of America; 3 Independent Researcher, Boston, Massachusetts, United States of America; 4 The Santa Fe Institute, Santa Fe, New Mexico, United States of America; 5 Department of Anthropology, Paleoecology Laboratory, University of Michigan, Ann Arbor, Michigan, United States of America; 6 PaleoResearch Institute, Inc., Golden, Colorado, United States of America; The University of Tulsa, UNITED STATES

## Abstract

The Belson site is located on an outwash plain draining the Early Algonquin stage of the central Great Lakes (coinciding with the Older Dryas stadial period around 14,000 Cal B.P) southwest across Lower Michigan into the Ohio tributaries. By 13,000 Cal B.P the St. Joseph River had incised multiple channels into this plain. On a terrace just north of a now-abandoned channel, a detailed surface study by Talbot from 2005–2018 showed several flake clusters largely of Attica chert, procured about 235 km southwest of Belson. A study of the surface sample was published by the authors in 2021 and indicated that the points were made with the Clovis technological pattern. Excavations in 2020–21 revealed hundreds of buried flakes and multiple tools in the lower, less-disturbed terrace sediment. Plotting of this material indicates successive occupations below the ploughed deposit and covering more than 30 *m*^2^. The buried assemblages are similar to the published surface assemblage with the addition of more small scrapers and manufacturing debris. Several of the buried tools have traces of proteins from a range of mammals, suggesting a broad-spectrum subsistence strategy. The documentation of a succession of little disturbed deposits with precisely recorded micro-debris will allow for testing of models describing settlement choice and developing dynamics of internal site organization. Initial analysis of recovered data provides support for an ‘outcrop centered’ model where high-quality chert outcrops serve as central places on the landscape. Samples of sediment and charcoal for identification and dating await study.

## Introduction

Studies of Late Pleistocene Clovis people in the Americas have successfully addressed the broad issues of chronology, environmental context, resource selection, mobility, and technological structure [1–22]. It is difficult to continue making progress on problems of resource movement between distant settlements, complexity of variation in technological systems, settlement organization, and local adaptation to new environments without new kinds of sites and new methods to monitor variation. The Belson site project is dissecting an unusual site with modern methods designed to answer such processual questions. This site rests on an outwash plain which drained the Early Algonquin stage of the central Great Lakes southwest across Lower Michigan and into Indiana tributaries of the Ohio River during the Older Dryas stadial period (ca.14,000 Cal B.P). By 13,000 Cal B.P the St. Joseph River had incised multiple channels into this plain which periodically flooded interfluvial areas covered by open meadows with scatters of spruce, fir and other trees and bushes, and traversed by mastodon, mammoth, bison, caribou and other animals [23]. On a terrace just north of a now-abandoned channel, there are several clusters of fluted points and fragments, scrapers, gravers and other tools, largely of Attica chert procured 235 km southwest of Belson [23]. Technological assessment of the surface sample indicates that the points were made with the Clovis technological pattern very similar to that described by Bradley *et al*. [14,23]. Excavation in 2020–21 revealed buried artifacts in the terrace sediment below the plow zone, which was largely undisturbed except for limited bioturbation from trees and rodents. Plotting of the chert items as small as a millimeter in this deposit indicates successive undisturbed occupation zones below the plowed deposit and covering 15 to 30 *m*^2^. These are similar to the published surface assemblage with the addition of more small scrapers and manufacturing debris. Additionally, several of the tools have traces of proteins from a range of mammals. This indicates a more diverse subsistence than is traditionally attributed to users of Clovis technology, supporting the Cannon and Meltzer [15] model for broad spectrum foraging within regionally variable environments. The documentation of a succession of little-disturbed deposits with precisely recorded micro-debris allows us to begin testing models of resource procurement, technological organization, and adaptation to new local environments, and settlement organization with new data, such as those proposed by Miller *et al*. [24], and Gardner [25,26].

The central Great Lakes region of Midcontinent North America has a long record of the study of Late Glacial foragers involving survey [27,28] and excavation [29,30]. Like much of North America east of the Plains, however, our Late Glacial sites are surficial and often disturbed, with little sediment cover to protect archaeological contexts, and consequently with little preservation of faunal and floral remains. Central Great Lakes archaeologists have become adept at extracting the maximum amount of information from disturbed and weathered stone tool assemblages [31–37]. Since the Great Lakes were still partially ice covered and the environment was harsh, it is not surprising that most of the sites of fluted point foragers have relatively late fluted point assemblages. Recently, however, several sites in the Great Lakes region have been reported that appear to manifest early fluted point assemblages: Rogers in south-central Ontario [38], Paleo Crossing in northern Ohio [31,39,40], Sheriden Cave in northern Ohio [41–43], Palmer in southeast Michigan [44,45], and Belson in southwest Michigan [23]. Like most Late Glacial sites, Palmer has cultural material only in plow disturbed deposits but Belson, to our surprise, has significant amounts of Late Glacial tools and debitage in less disturbed sediment below the plowed zone.

This situation required us to recover all identifiable cultural material from the plowed deposit, and carefully plot cultural items in three dimensions below the plowed zone with a Total Data Station (TDS). This will allow us to test different propositions about the degree and the mechanisms of disturbance and the existence of surfaces or zones of accumulation. Before presenting the results of this procedure and our preliminary studies of the artifacts recovered, some additional background on the project will be given.

## Excavations in 2020–2021

### Discovery and initial research

**Fig 1:**
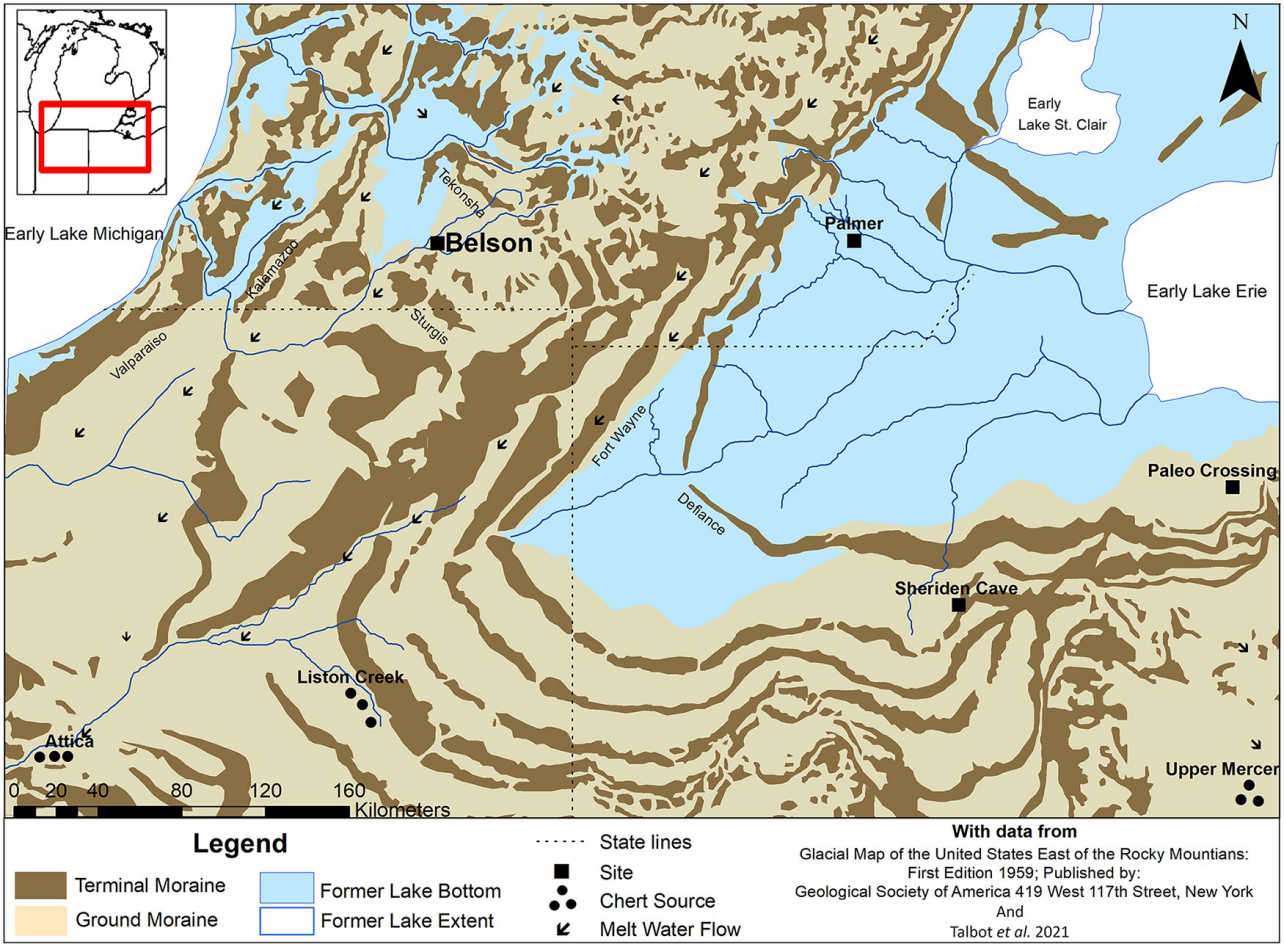
Map of early Great Lales paleo sites with key periglacial features and stone sources.

The Belson site is situated on the terrace of a Late Glacial channel of the St Joseph’s River which drained glacial meltwaters from much of Lower Michigan (Fig 1). In the open spruce-fir parkland, people who camped on the terrace would have had a good view of herds approaching the channel and crossing from the south. During the Holocene, however, the site was situated between rich marsh and pond areas and upland Oak-Beech-Hickory Carolinian forests, and not surprisingly has Archaic and Woodland occupations. In 2008 Talbot found a concentration of Late Glacial fluted bifaces and flake tools, all on the distinctive Attica chert from West-central Indiana [23]. A few tools might be from lost tool kit or cache, but a visit to the site by Talbot and Wright in 2017 yielded not only finished formal tools, but small flakes of Attica chert indicated that there had been occupation here. At this point, we commenced a study of the surface finds. Apparent affinities with Clovis assemblages led Talbot and Wright to enlist Nash, who had years of experience with such assemblages from Texas. Nash quickly recognized details in biface reduction characteristic of Clovis assemblages as described by Bradley *et al*. [14]. A text illustrating the material and initial theoretical implications was published in *PaleoAmeric*a [23].

### Materials and methods for the excavation

**Fig 2:**
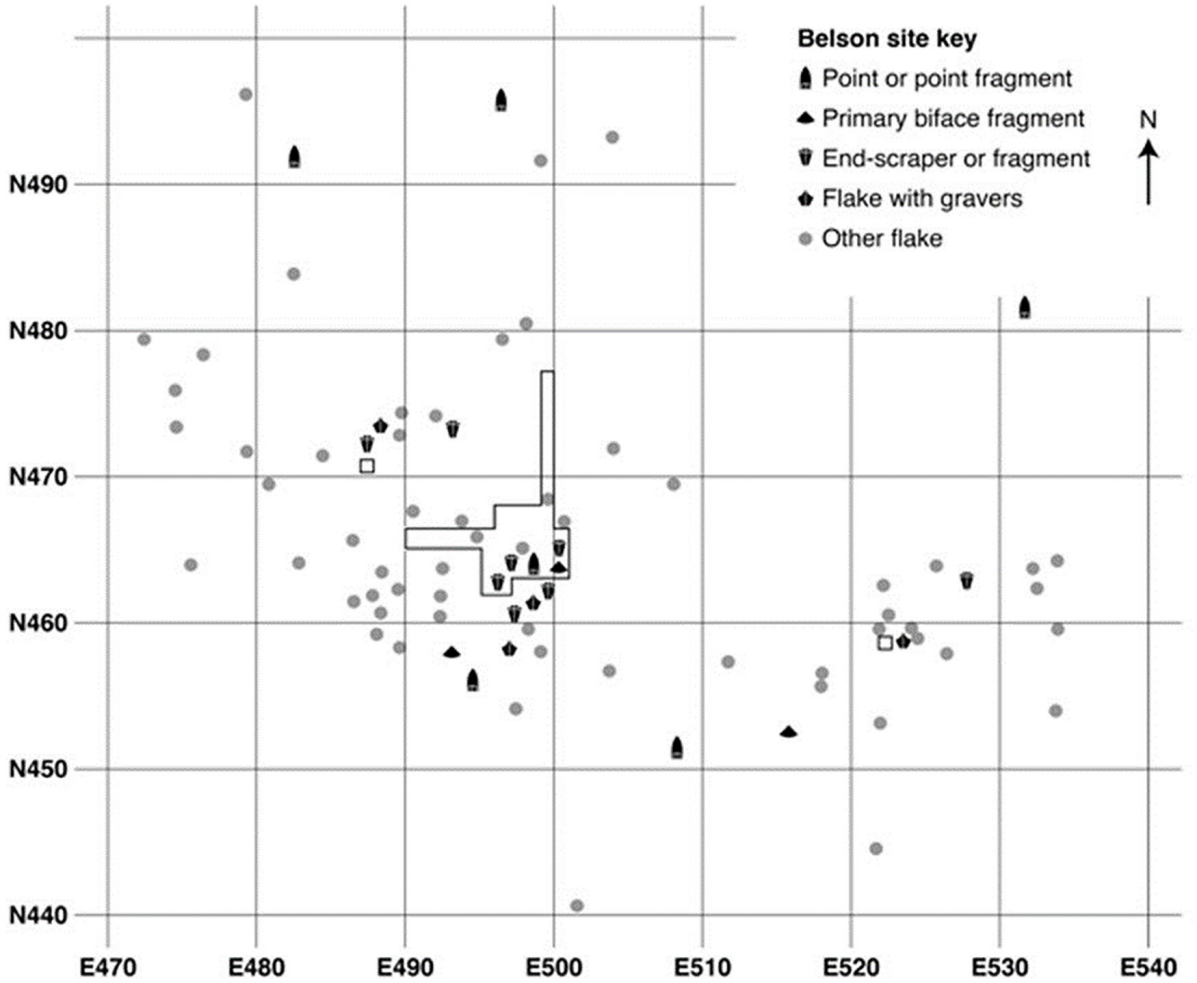
Surface and plow zone artifacts of Attica chert found prior to 2022.

There are at least three clusters evident in the distribution of surface finds (Fig 2) aligned from southeast to northwest. In 2019, Test Unit #1 in the southeast cluster yielded a flake with graver spurs *in situ* below the plow disturbed deposit. Subsequent test units in the central cluster had a few small flakes also in seemingly undisturbed deposits. Based on these results, we decided to conduct a precision excavation of the deposit, screening all sediment, from both the plowed and sub-plow deposit with one 1/8^th^ inch mesh, retaining all identifiable cultural material. The excavation was conducted on private land and no permits were required for the described study.

For the sub-plow deposit, we hand excavated in..50 m quadrats, and..05 m levels, plotting each flake, charred item, and all other items of note in three dimensions with a TDS. Any artifact with potential for residue analysis was, after plotting, placed un-touched in a sterile container with samples of the surrounding sediment. All quadrants were excavated and screened to a depth of about 99.25 m above datum or..60 m below the surface. After that, for units excavated in 2021, another..05 –.10 m of sediment was shoveled out and sieved with one 1/8 inch mesh to make sure that we reached the bottom of the cultural deposit. This final step produced little cultural material, indicating that excavating this depth in 2020 likely recovered most of cultural items. Each possible disturbance of the sub-plow deposits was excavated separately. All of these were judged to be Holocene animal burrows or tree stumps and roots, with the exceptions of the disturbance termed ‘Feature 1’ (discussed below), which may be a Late Glacial pit or hearth.

### Results of the excavation

#### Excavations in 2020

**Fig 3:**
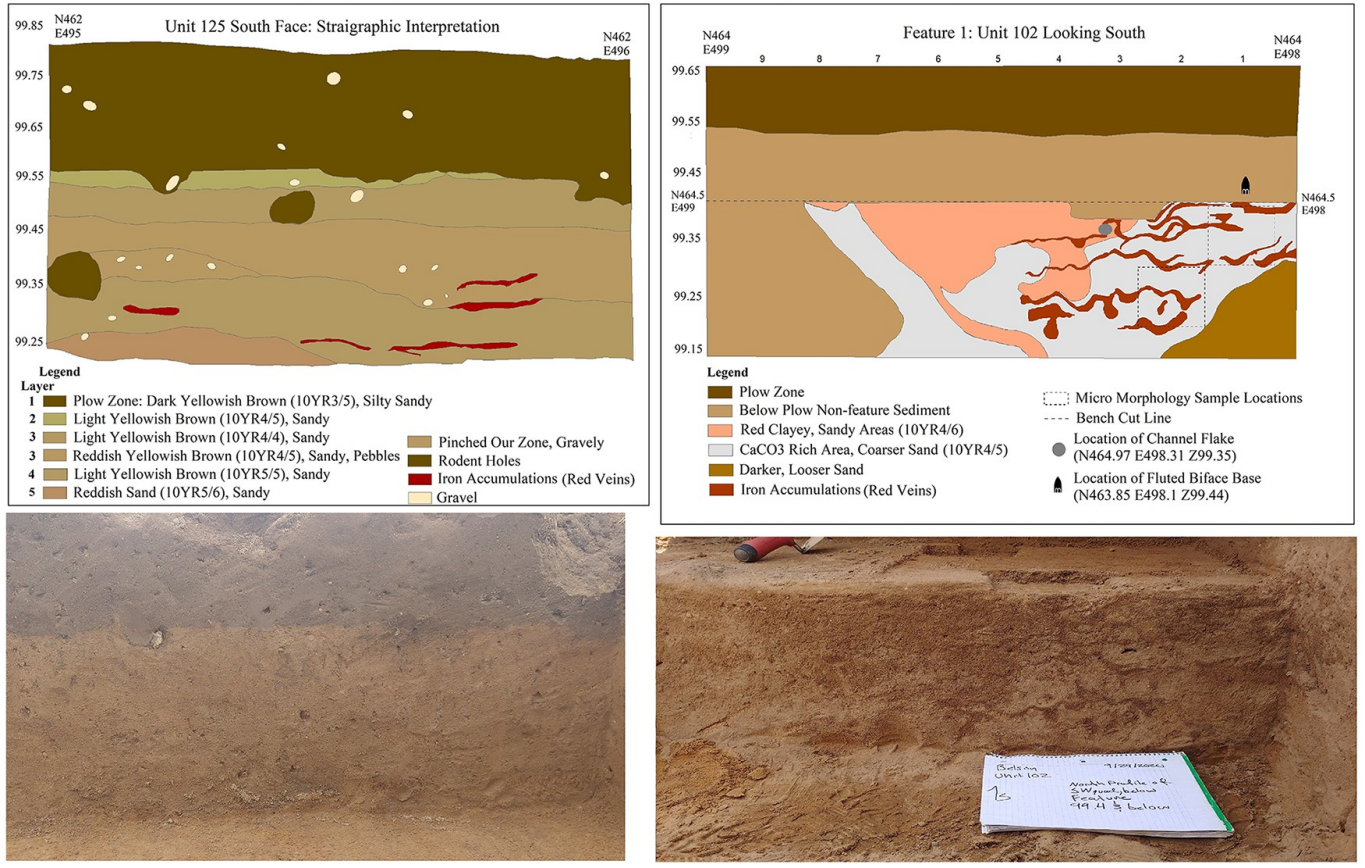
Geological profile and Feature 1. (Left) Stratigraphic interpretation and associated photograph. The color interpretations are exaggerated to depict subtle color differences noted in the field, which do not show well in the photographs. See Munsell colors in the legend for precise color determinations. (Right) Interpretation and photograph of east-west bisection of Feature 1 during the excavation in September of 2020.

During the 2020 excavation season we finished a total of 31 one-meter squares. First, we hand excavated a row of 10 units north-south on the East 500 m gridline and a row of 10 units east-west on the North 465 m gridline, trying to transect the central cluster of flakes evident on the surface (Fig 2). For additional photos of the 2021 excavation see supplemental information (S1–S3 Figs). A substantial scatter of flakes was encountered in the deposit beneath the plowed zone (see section *Spatial analysis of the excavated flake cluster)*. After excavating the two 10 m long trenches by hand, we encountered a possible Late Glacial cultural feature—referred to as Feature 1— (Fig 3 right) when excavating additional, abutting units to the north and west of the two trenches. In areas that were not associated with possible cultural features, the sediment undisturbed by modern ploughing contains similar stratigraphic sequence, with limited bioturbation, throughout the excavation block (Fig 3 left).

Feature 1 was a small, relatively deep pit first noted at an elevation 99.40 m above our arbitrary datum, and about..15 m below the base of the plow zone (Fig 3 right). It was about 1.70 m N-S and 1.85 m E-W. It is irregular in shape and features a central depression with a concave bottom that reached a depth of about 99.25 m above datum (Fig 3 right). At its base the fill was pale brown sand in contrast to the surrounding light reddish sand. The feature material had a notable concentration of small Attica chert flakes that occurred deeper and in greater density than in the surrounding units. Two diagnostic artifacts were recovered from the feature area *in situ*. One is a partly fluted and broken biface fragment (see Fig 7D in the *Flaked stone tools* section), which was pot-lidded by burning after it was broken. This artifact was recovered near the top of the feature standing on its edge (rather than flat lying) resting against the inside wall of the feature. The other is the distal part of a channel flake recovered from the central portion of the lower part of the feature lying relatively flat (see Fig 7G in the *Flaked stone tools* section).

The feature fill had a scattering of charcoal, now being studied. Our preliminary assessment is that this feature was a dug-out heating feature that was subsequently filled in by natural processes with the surrounding sediment that contained a high concentration of exclusively Late Pleistocene debitage. This would explain why only 9.13% of the flakes within the feature had evidence of heating, which is similar to the percent of heated flakes from the surrounding areas of the cluster where 9.76% had traces of heating (S10 Fig). After filling in, subsequent soil leaching process including the downward migration of iron oxide minerals and clay particles caused the mottled clayey and sandy patches that comprise the fill. Prominent sinuous iron oxide features (red veins) that crosscut these areas are likely to result from more recent ground water movement in the feature area. See supplemental information for additional photos of the Feature 1 excavation (S5–S8 Figs).

Table 1 describes the stratigraphic units from top (later) to bottom (earlier) as recognized in excavation wherever there was little disturbance by cultural features or natural disturbance such as tree stumps or rodent burrows. The soil is characterized as “Bronson Sandy Loam” in the detailed study of Cowan [46]. The elevations show little variation in the excavated area, but this may change with further excavation. This is a provisional archaeo-geological discussion which must be further evaluated with future work.

**Table 1:** Provisional archaeo-geological description of strata as recognized in excavation.

Stratum	Description
**1**	The plow zone, from the surface to about..35 m below the surface, and about 99.85–99.55 m above arbitrary datum is a result of the recent mixing of the humic A_**H**_ and most of the leached A and horizons by repeated ploughing and all the cultural material deposited in them in Archaic, Woodland and Recent times. The USDA soil survey refers to this as the Ap horizon [46: p.49].
**2**	From about..30-.37 m below surface and 99.55–99.48 m above arbitrary datum is a very light-yellow sand from which iron minerals have been leached. At present this is visible primarily in the ridges between plow scars. This sand was probably more than 16 cm thick, but most of it has been mixed with the humus layer to form the present plow zone. Given the near absence of pebbles, it is possible this sand is in part carried in by wind, perhaps in latest Pleistocene Younger Dryas times [47: p.177-79]. The leaching, however, would be a Holocene soil weathering phenomenon producing an A_**L**_ or E horizon.
**3**	From about..37-.60 m below surface, and 99.48–99.25 m above arbitrary datum there is a fairly homogenous sand with subtle color differences between the light yellowish brown upper portion and a reddish yellowish brown lower portion and has small sub-round to round pebbles. Based on the distribution of flakes, we recognize several successive occupational zones within this deposit (discussed below). The USDA soil survey denotes this horizon as the B21t horizon [46: p.49].
**4**	From about..60-.75 m below surface and about 99.25–99.10 m above arbitrary datum, we found a light brown sand with clearly defined lenses of light reddish-yellow clayey sand and small sub-angular to sub-round pebbles. We interpret these as overbank flood deposits with different velocities. Human traces such as tiny flakes are less common in these deposits. The USDA soil survey denotes this horizon as the B22t horizon [46: p.49].
**5**	About..75 m below surface and below, around 99.10 m above arbitrary datum, we have observed a homogenous light brown sand without significant clay or silt components but with rare small pebbles. We have excavated and screened little of this. The USDA soil survey denoted this horizon as the B23t horizon [46: p.49].

#### Excavations in 2021

**Fig 4:**
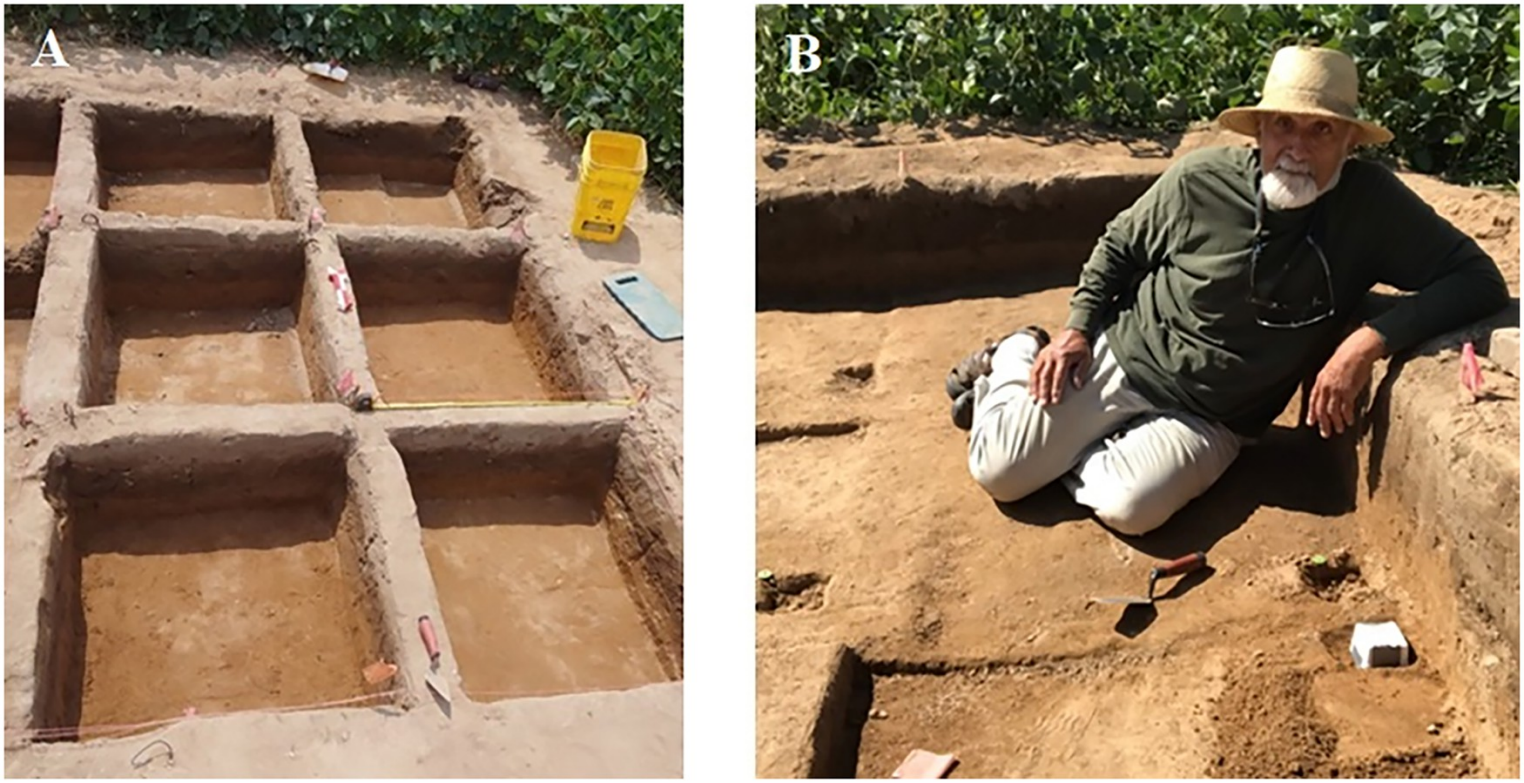
Excavation methods. (A) 2021 Excavation showing Block and Baulk technique to conserve sections and find faint features. (B) Herny Wright taking micro-morphological samples after removal of the baulks. The individual pictured in Fig 4 has provided written informed consent (as outlined in PLOS consent form) to publish their image alongside the manuscript.

During the second season of excavation in 2021, we better defined the east edge of the central cluster but did not reach the south or west edges of the cluster. Because of the greater number of items requiring measurement with the TDS, we completed only 16 one-meter units. During this season, we introduced a block-and-baulk system (Figs 4A and S4), excavating a..90 -..85 m block down to 99.44 m above arbitrary datum and leaving a..10 -.15 m balk on two sides to facilitate the drawing of sections. In each one-meter square, one randomly placed 6 x 6 x 4 cm sample was removed from about 99.44–99.40 m above arbitrary datum—the level at which we subjectively detected a concentration of small flakes—for future micro-morphological study when funds are available (Fig 4B). We realize that this process may result in less complete observation of small items, as some may be contained within the micro-morph samples. We hope that the benefits of the analysis in better defining activity surfaces will outweigh the losses. After micro-morphology sampling, we hand excavated the units to a depth of about 99.20 m above arbitrary datum. As we moved southwestward during this second season, we found more scrapers and flakes related to scrapers than in season 1. It is likely we are entering an interesting new activity area. Analysis shows a deeper, relatively dense distribution of flakes to the southwest, which we tentatively term ‘Feature 2’.

## Flaked stone assemblage from the central cluster

### Raw materials and long-distance procurement

The majority of the flaked stone items from the Late Glacial contexts at the Belson Site are made on Attica chert [48: p.90-99, 49: p.11-18], found as tabular pieces in outcrops near Independence, Indiana, 235 km southwest of Belson. We thank Brad Koldehoff for giving us geological samples from the Attica source. The original finds from Belson were very kindly identified by Tony DeRegnaucourt as three varieties of Attica (Table 2).

**Table 2:** Three Attica chert varieties described by Tony DeRegnaucourt and Tom Talbot.

Attica Variant	Description of chert
**White/green**	Banded white/green and tan chert, colloquially termed “Indiana green” or “Wabash green” the white being chalcedonic chert and the green being white chert with particles of glauconite.
**Blue/gray**	Blue/gray chert, the primary component being fine grained chert making it more translucent. There is much iron oxide in the form of limonite and some pyrite particles. Little glauconite is evident in this variant.
**Gray/tan**	Banded gray/tan chert, the gray being chert with some glauconite and the tan being chert with particles of iron pyrite.

Fossil sponge spicules occur in all variants. No other fossils were noted. All variants have examples with fine texture on flake surfaces; the first two variants also have examples with medium textures and matte surfaces. All stages of utilization of Attica chert from primary flakes and biface fragments to finished and broken tool fragments are represented at Belson.

A small minority of the chert items are made on Liston Creek chert [49: p.54-55, 48: p.132-133] found as tabular pieces in outcrops southeast of Peru, Indiana, 155 km south of Belson. This is a homogenous white to light gray chert, sometimes with amorphous gray mottling. Fossils and microscopic crystals have not been observed.

A tiny proportion of the small flakes and a few tools are made from other cherts probably from distant sources. A few small flakes have been visually identified as Flint Ridge or Vanport chert and Coshocton or Upper Mercer chert, both from East Central Ohio. One artifact, a thinning flake from a secondary biface [*Sensu* 50] of a fine translucent banded tan chert, originally ascribed to Flint Ridge, is precisely duplicated by a flake from DeRegnaucourt’s [48: Fig II F] Upper Mercer collection. These sources are both about 350 km southeast of Belson. A point base broken during fluting (see Fig 7D in the *Flaked stone tools* section) is identifiable as Paoli chert from north-eastern Kentucky based on distinctive microscopic hematite particles.

Only a few similarly small items, plus one triangular end-scraper from the surface (see Fig 7C in the *Flaked stone tools* section), are made from cherts available locally. Most recognizable among these is Constantine chert, a fine grained, mottled white, gray, and blue chert. It contains cryptocrystalline, microcrystalline and chalcedonic quartz. Fine blue lines are veins of embedded quartz. There can be small amounts of glauconite present, though the large amount often found in Attica is not observed. Constantine chert can be found on the surface in small irregular blocks on a sand deposit around seven km northwest of Constantine, Michigan, about 30 km from Belson. It also can be found in the form of small pebbles in the glacial till and outwash throughout the area.

#### Discussion of raw material procurement

To date, we have found no core fragments, or cortical flakes from the trimming of cores. It appears that the Belson Late Glacial stone knappers had come directly from the Attica quarries, carrying large flakes struck from cores, perhaps already retouched for use as heavy cutting or scraping tools (see Fig 8C and 8E in the *Flaked stone tools* section), or primary bifaces made from such flakes (see Fig 7A–7C in the *Flaked stone tools* section). The few flakes of other exotic stones struck from secondary bifaces (see Fig 8 F in the *Flaked stone tools* section), or refurbishing finished points imply only that the Belson people had indirect contacts with other distant quarries. The local cherts probably come from pebbles eroding from till or outwash, and not from bedrock quarry sources. The diversity and amounts of items on Attica chert imply procurement from the distant source. This pattern of long-distance provisioning with Attica chert already reported from Muller-Keck, 320 km West-Southwest of the source [51] and from Palmer 330 km East-Northeast of the source [45], and The Attica source is near the centroid of these three sites. This does not support the proposal that high quality stone sources were discovered as people making Clovis tools initially moved west and north [52] and were perhaps revisited because of their superior quality. A central location better fits the proposal that groups were tethered to centrally located sources [24,25]. Given the many high-quality cherts available in central and southern Indiana [48,49] it is a question why the Attica source was preferred. A possibility for future consideration is that this striking, typically green silicate was useful in social signaling.

## Spatial analysis of the excavated flake cluster

### Materials and methods for the spatial analysis

This analysis of the excavated lithic material will focus on the two main variants of Attica chert that predominate the deposit: blue/gray and white/green. The Attica chert is associated with the fluted bifaces as no diagnostic artifacts from the site—other than 7 of the 8 fluted bifaces—are made from it. Moreover, Attica flakes predominate in the lowest cultural layers at the site, which also contained a broken, fluted biface made from Attica chert (see Fig 7E in the *Flaked stone tools* section). See the S1 Dataset for all of the flake data, and associated specimen numbers, used in the spatial analysis. The specimens in this study are privately held and were studied on loan to the University of Michigan Museum of Anthropological Archaeology (UMMAA) in Ann Arbor Michigan USA and will be made accessible upon request through UMMAA.

An analysis using Chi Square tests will consider the statistical significance of the pattern of flake distribution between cultural zones, and between the plowed and sub-plow deposit. Comparisons of flake weight and material type within and below the plowed deposit will also be considered. These data will be used to assess various differences in deposition and distribution between the two main Attica chert variants, which document the dynamics of forager settlement use, indicating successive Late Glacial visits to the sites.

In the *Distribution and mapping of below-plow deposit* section, we have attempted to separate the flakes from bifacial refurbishment from scraper refurbishment to better understand any differences in activities by area of the site. These determinations are based largely on platform angle and flake morphology, with flakes from bifaces having much smaller platform angles, and flakes from scrapers having morphologies that mirror the distal edge of end-scrapers.

### Results of the spatial analysis

#### Summary of the data for spatial analysis

From the 40 1 x 1 m units excavated in 2020 and 2021 that contained lithic material, a total of 1,256 flakes were recovered. 618 (49%) flakes were recovered from below the plowed deposit, while 638 (51%) flakes were recovered from within the plowed zone. 964 (77%) of the flakes were determined to be on Attica chert by Talbot, of those, 535 (56%) flakes were recovered from below the plowed deposits, while 428 (44%) were recovered from within the plow zone. The Attica comes in three varieties: blue/gray; white/green; and gray/tan. The total number of blue/gray Attica flakes is 537 with 241 (45%) occurring below the plow zone and 295 (55%) within. The total number of white/green Attica flakes is 416 with 285 (69%) occurring below the plow zone and 131 (31%) above. The gray/tan variety totals only 11 flakes with 9 occurring below the plow zone and 2 within. The mean weight of flakes in the Attica assemblage is..092g. The mean weight of the blue/gray and white/green variants are..094g and..084g respectively, while the mean weight for the non-Attica flakes is..14g. The mean weight of Attica flakes from the below plow deposit is..089g, while the mean weight for Attica flakes from within the plow zone is..096g. The mean depth of flakes below the plow zone is 99.452 m above arbitrary datum for the blue/gray variant and 99.441 m above arbitrary datum for the white/green variant (Table 3).

**Table 3:** Summary of excavated flake assemblage data.

Material Variant	Total Count and Mean Weight	Plow Zone Count and Mean Weight	Below Plow Zone Count and Mean Weight	Mean Depth for Flakes Above Arbitrary Datum
**Non-Attica**	292 (.487g)	210 (.622g)	82 (.14g)	99.451 m
**All Attica Variants**	964 (.092g)	428 (.096g)	535 (.089g)	99.446 m
**Blue/gray**	537 (.094g)	295 (.086g)	241 (.103g)	99.452 m
**White/green**	416 (.084g)	131 (.119g)	285 (.06g)	99.441 m

In sum, these data indicate that about half of the total recovered flake assemblage remained largely undisturbed from modern plowing. Over three quarters of the total flake assemblage is represented by Attica chert associated with the Clovis bifaces, and more than half of the Clovis deposit remained undistributed by modern plowing.

That the white/green Attica variant is deeper on average than the blue/gray, and that nearly 70 percent of the white/green flakes are below the plow zone, while only 45 percent of the blue/gray flakes are from below the plow zone, suggesting that activities involving the white/green flakes came before activities involving the blue/gray flakes. When considering the absolute counts of the two Attica variants from within and below the plowed deposit using a chi-square test, the difference is significant beyond the..0001 level (S1 Appendix).

The large difference between the average weight of flakes in the Attica assemblage relative to the flakes on other materials is likely the result of differences in activities being performed. The Attica assemblage likely represents refurbishment or resharpening of broken or worn-out tools. Considering the distance from the Belson site to the Attica source (235 km), it is likely that these tools had been previously refurbished with the size of the tools and the associated refurbishment debitage trending smaller as the distance from the source increases.

Downward movement of very small flakes through bioturbation, and the higher likelihood of a plow contacting relatively larger items is potentially a cause for the slight difference in mean flake size between Attica flakes in the plow zone versus the below plow deposits (Table 3). However, the trend in flake weight (as an approximation for size) does not decrease significantly with depth, indicating that downward movement of flakes is limited (S9 Fig). Furthermore, fire-cracked rocks commonly produced in boiling, steaming, and cooking during Holocene times are common in the Plow Zone (367 pieces weighing a total of 10,476g), but very rare in the below plow deposits (only 5 pieces weighing a total of 151.5g recorded). This is a strong indication that movement of cultural items through the soil column is limited.

#### Distribution and mapping of the below-plow deposit

Many of the below plow flakes were found *in situ* and piece plotted to that location (n = 155), while others were recovered through the one 1/8^th^ inch screen from bracketed elevations within a..50 x..50 m quadrant of a unit (n = 463). Flakes recovered from the screen were assigned to the three-dimensional center of their quadrant for the spatial analysis that follows. Moreover, flakes collected in the first few days of excavation in units N465-E497, E498, and E499 were not collected in quadrants (n = 24), nor were flakes in unit N464 E498 (n = 23). The former three units were originally excavated down to an elevation of 99.45 m above arbitrary datum, while the latter unit was taken down to 99.50. Each of these units were taken to a lower elevation of 99.25 m above arbitrary datum later in the 2020 season, where flakes were recovered in quadrants and *in situ*. For the spatial analysis, flakes not recorded to the quad level or piece plotted, were scattered relatively evenly throughout the 1x1 meter unit within the elevation brackets which they were recovered.

**Fig 5:**
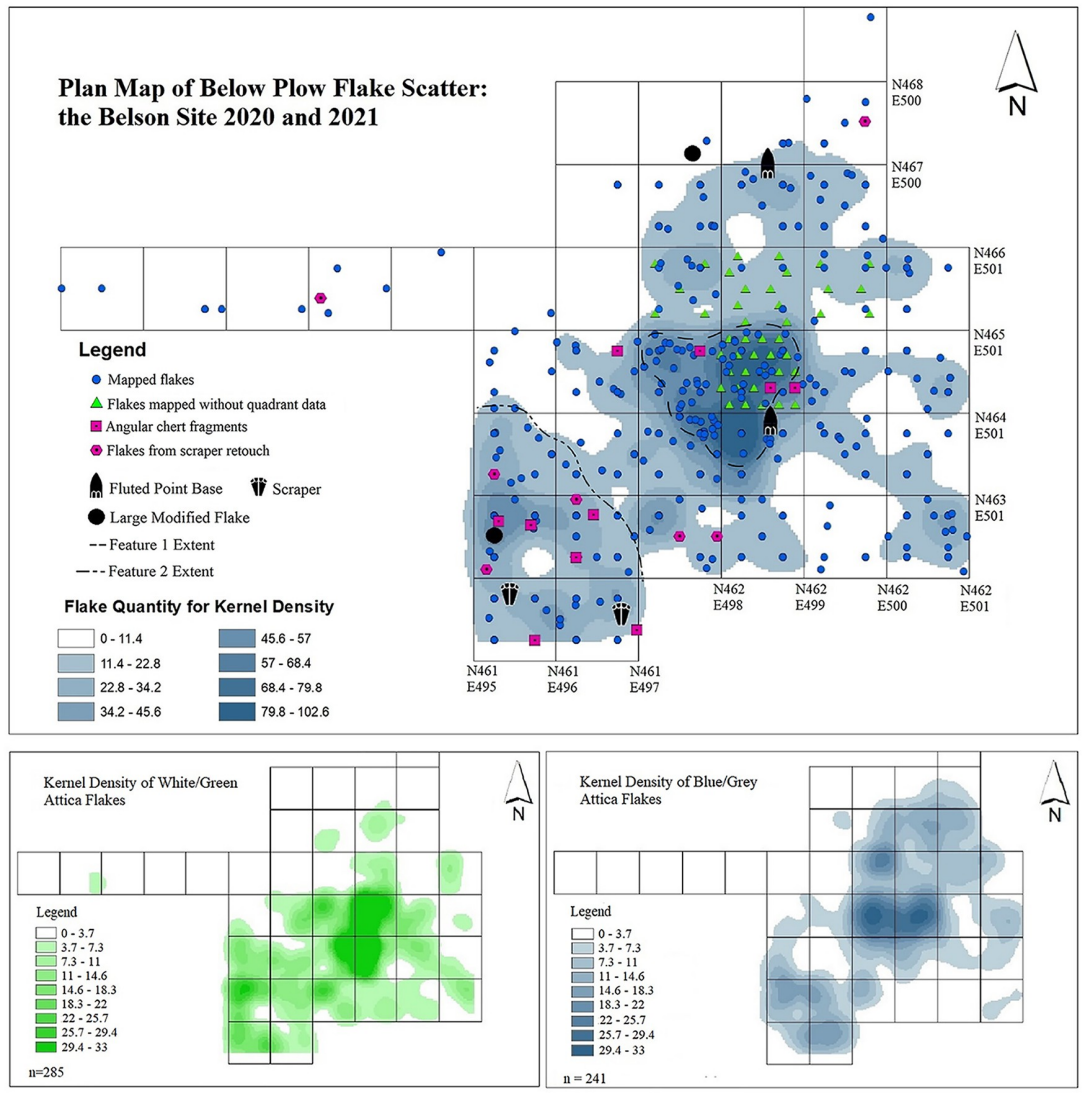
Plan view of below plow flake cluster. (Top) A plan view plot of flakes recovered from below the plow zone during the 2020 and 2021 excavation seasons. The location of flakes collected from the screen, which are assigned to the center of the quad where they were recovered, while piece-plotted flakes are placed at their find location. Flakes not plotted to the quadrant level in the upper part of four units (N464 E498, and N465 E499, E498, E497) (green triangles) are distributed relatively evenly throughout the 1 x 1 meter unit within measured elevation brackets, as per observations in the field. The kernel density data indicates flake counts for specific areas. (Bottom) Kernel density in plan view of the two main Attica variants. The kernel density maps used to illustrate the clustering of flakes in this portion of the analysis were created with ArcGIS 10.8.2 Spatial Analyst Kernel Density tool.

In plan view (Fig 5), flaked stone items in the below plow zone deposit are scattered throughout the excavation but form a central concentration associated with Feature 1, with a less dense put possibly larger concentration of flakes occurring in the southwest portion of the excavation tentatively referred to as Feature 2.

All of the recovered flakes with the necessary observable attributes have been assessed technologically to determine if they derive from bifaces (n = 1,051, 578 from plow zone and 473 from below the plow zone), or end-scrapers (n = 21, 13 from the plow zone, and 8 from below plow). Note that refurbishing a biface produces many more flakes than refurbishing an end-scraper. Also, our criteria for identifying scraper retouch flakes are still developing. Thus, the differences in count of flakes from the two tool classes does not necessarily indicate differences in intensity of activities performed at the site. An “angular” classification (n = 29, 20 from the plow zone, and 9 from below the plow zone) has been given to flakes where the technologically is indeterminable, due to blocky, non-flake like morphology. Angular fragments are suggestive of activities not related to the relatively fine bifacial refurbishment and resharpening that was occurring at the Belson site, evidenced by the small mean size (weight) of Attica flakes struck from bifaces.

The distribution of knapping debris and formal tools not associated with bifacial reduction indicate that in the southwest portion of the excavation, associated with potential Feature 2, different classes of tools and associated activities are being represented (Fig 5). Although alone the large amount of biface refurbishment flakes would likely be interpreted as representing a short-term hunting camp, the presence of scrapers and their refurbishment debris indicates that other activities related to processing were taking place at the site during the Clovis occupations.

When divided into the two main Attica variants, we see different but overlapping distributions in plan view (Fig 5 bottom). The white/green variant tends to occur low in Feature 1 and predominantly to the southwest, while the blue/gray variant tends to occur higher in Feature 1 and predominantly to the northeast, with some occurring alongside the white/green variant in the southwest concentration. As discussed below, the vertical distribution likely indicates two phases for the use of Feature 1.

**Fig 6:**
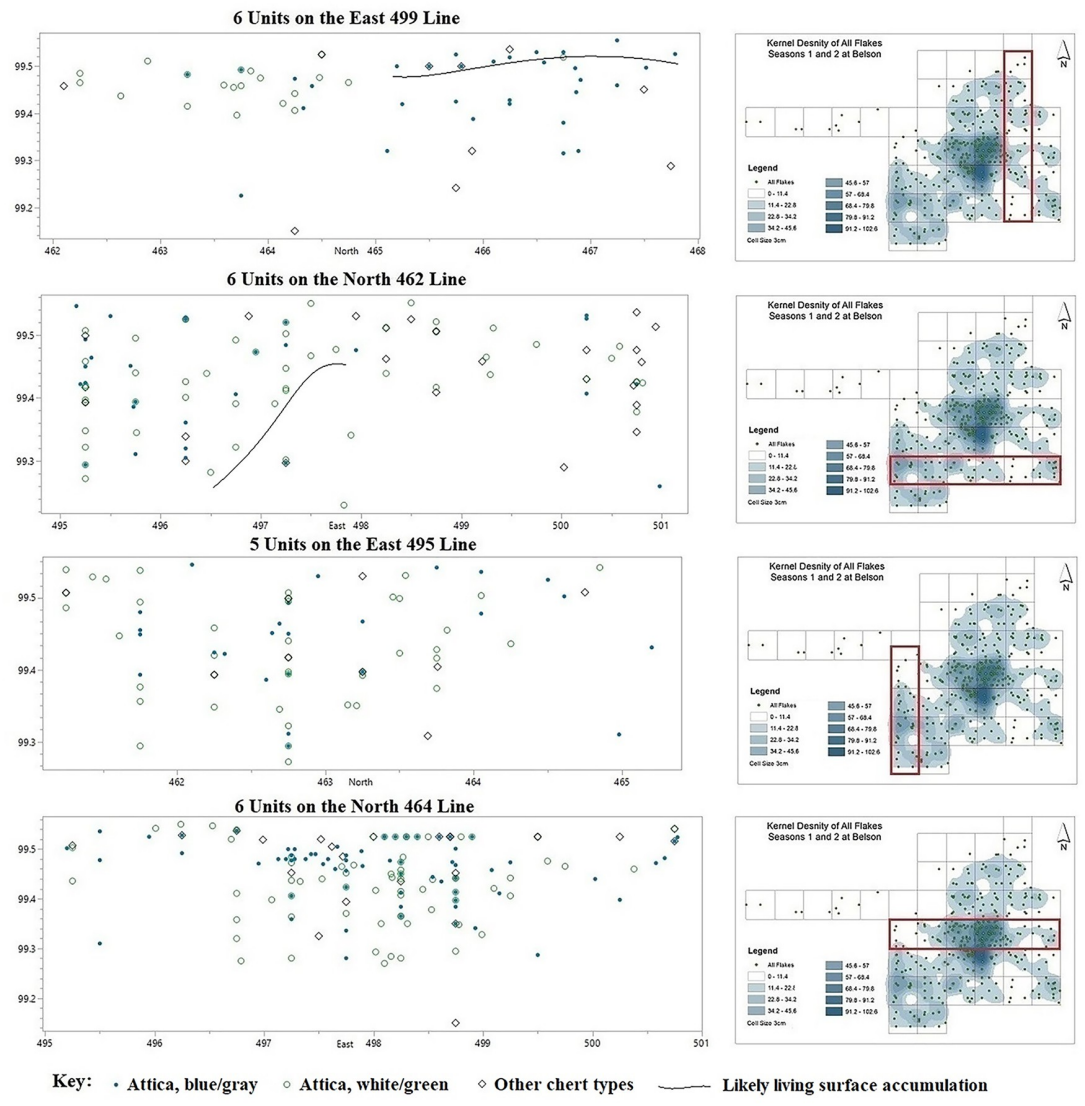
The occurrence of below-plow flakes in one-meter squares on select transects. Shows the structure of the sub-plow deposit with respect to the differing flake types.

Vertically, the Attica flakes occur within the first 40 cm below the plow zone and indicate two bowl-shaped depressions of differing sizes (i.e., Features 1 and 2). The three-dimensional occurrence of flakes from four transects of 1x1 meter units through the excavation block have been modeled as back-plots to show the structure of the deposit below the plow zone, focusing on the two different Attica chert variants (Fig 6).

Flake distribution from the six units on the East 499 line which does not intersect directly with Feature 1 or 2, provides evidence for the vertical distribution of artifacts avoiding complications associated with the pit or dug-out feature. This plot shows that the majority of flakes occur between 99.55–99.40 m above below arbitrary datum with a smaller amount occurring between 99.40–99.25 m above arbitrary datum in the northern units. There is also a likely surface accumulation of blue/gray Attica flakes around 99.50 m above arbitrary datum on the northern three units. Importantly, there is a clear separation between the white/green flakes in the southern three units of the transect at a slightly lower elevation, and the blue/grey flakes in the northern three units at a slightly higher elevation.

Flake distribution from the six units on the North 462 row shows the east part of a bowl-shape depression with a bottom around 99.27 m above arbitrary datum in the western two units, which we term Feature 2, while the eastern most unit is predominated by non-Attica flake types. It appears that there is a surface accumulation of white/green flakes on the edge of Feature 2 leading to the bottom. This supports the idea that the white/green flakes were deposited before the depression filled in with the mix of blue/gray, white/green, and other chert types. This pattern suggests that activities involving the white/green chert took place before activities associated with the blue/gray and other chert types in this area, as in other areas.

Flake distribution from the five units on the East 495 line shows the same bowl-shaped distribution of Feature 2 as the western three units on the North 462 transect, but better defined in its north-south axis. In the lower portion of Feature 2 from 99.38–99.27 m above arbitrary datum between North 465–461 there are 13 white/green flakes and 4 blue/gray and others. In the upper portion 99.48–99.38 m above arbitrary datum there are 17 white/green and 21 blue/gray. A chi-square test yields a probability value of..029, indicating that the pattern of white/green flakes occurring below the blue/gray flakes is significant at the..05 level (S1 Appendix). The pattern of white/green flakes occurring in the bottom of the bowl-shaped feature seen in the North 462 row is confirmed by the occurrence of flakes on the East 495 transect and indicates that activities involving the white/green chert were more common than those involving the blue/gray flakes when this part of the excavated area was first utilized.

Flake distribution from the six units on the North 464 line shows the bowl-shaped occurrence of flakes associated with Feature 1 transecting the center of the excavation block. Similar to Feature 2, Feature 1 is lined exclusively with white/green, while a mix of blue/gray, white/green, and other chert types comprise the feature fill. In lower Feature 1, 99.38–99.27 m above arbitrary datum between East 499.5–496.5, there are 22 white/green flakes and 9 blue/gray and other. In the upper portion between 99.38–99.48 m above arbitrary datum there are 33 white/green and 34 blue/gray and other flakes. A chi-square test yields a probability value of..044, indicating that the pattern of white/green flakes occurring below the blue/gray and others in Feature 1 is significant at the..05 level (S1 Appendix). This is strong evidence that for activities associated with Feature 1, the white/green flakes characterize the first use of the feature, before extensive use of blue/gray and other chert variants.

### Conclusions for the spatial analysis of excavated material

Taken together, the distributional and technological data from our first two seasons of excavation indicate that the below plow deposit represents multiple visits to a Terminal Pleistocene habitation site, where activities related to the refurbishment of bifacial hunting weapons (fluted points), and material processing using triangular end scrapers and side scrapers took place. The differing distributions of the white/green chert versus the blue/gray chert likely represent at least two different early occupations of the site by users of Clovis technology. Plowing likely disturbed at least one additional later occupation.

That the two Attica sub-plow features show clear separation vertically strongly suggesting separate occupations, although the amount of time between them is unclear. They could represent a difference in time anywhere from a few weeks to a few decades. However, that the two assemblages are both of Clovis technology, use raw materials from similar sources, overlap significantly in space, and reuse the same pit features, speaks to a relatively short span of time between them. The white/green flakes lining the depressions of both concentrations represents the first identifiable occupation of the site, while the subsequent infilling with blue/gray, white/green, and other chert types represent some small number of successive occupations by Clovis technology users, perhaps just one or two. The Attica flakes in the plow zone may represent a mix of only the two identified occupations, or it may include one or two additional occupations. A more thorough examination of the three-dimensional distribution of cultural material will follow two more seasons of excavation. We will reassess the number of occupations represented by the Attica assemblages, and whether the different occupations represent similar uses of the site, or if the site function has changed through time.

Even with the present data from only part of the central cluster at Belson, we can consider issues of settlement choice and dynamics. The two features observed have similar depositional histories, with the same chert variant predominant in the first use each feature and diverse variants in subsequent uses. However, though the sample sizes are small, the tool debris found around these two features are different (Fig 5). Around Feature 1, biface fragments and refurbishing flakes predominate; around Feature 2, end-scrapers and their refurbishing flakes are more common. This pattern holds true for both occupations and shows that the forager group returned to a familiar campsite and performed activities in the same places. This is conformable to the expectations derived from Binford’s model of the continua of forager to collector site-use under different ecological conditions [53,54]. This continuity in settlement layout suggests a fixed strategy in the dispositions of logistical tasks. This is not the expectation when dealing with initial, short-term or exploratory use sites with limited activity performance. It appears that we have not yet found such an initial occupation at Belson, or such initial use leaves so few material traces that we cannot yet detect them with present evidence, or perhaps an initial exploratory use was not needed and this expectation must be modified or rejected.

## Flaked stone tools

The initial study of Talbot’s surface collections [23] was focused on a series of fluted bifaces and a few other items. We argued that the shallow basal concavities, small multiple flutes, and over-face bifacial thinning, indicate that the fluted bifaces at Belson shared technology with Clovis as described by Bradley *et al*. [14]. Since 2019, additional surface finds and artifacts recovered during the first two seasons of excavation have yielded a broader spectrum of tools, facilitating a *chaine opérètoire* analysis of production, use, maintenance, breakage, and re-cycling. For the specimens analyzed in this portion of the analysis, the figure identification numbers that appear in the text are used as the specimen numbers in the museum records at UMMAA.

**Fig 7:**
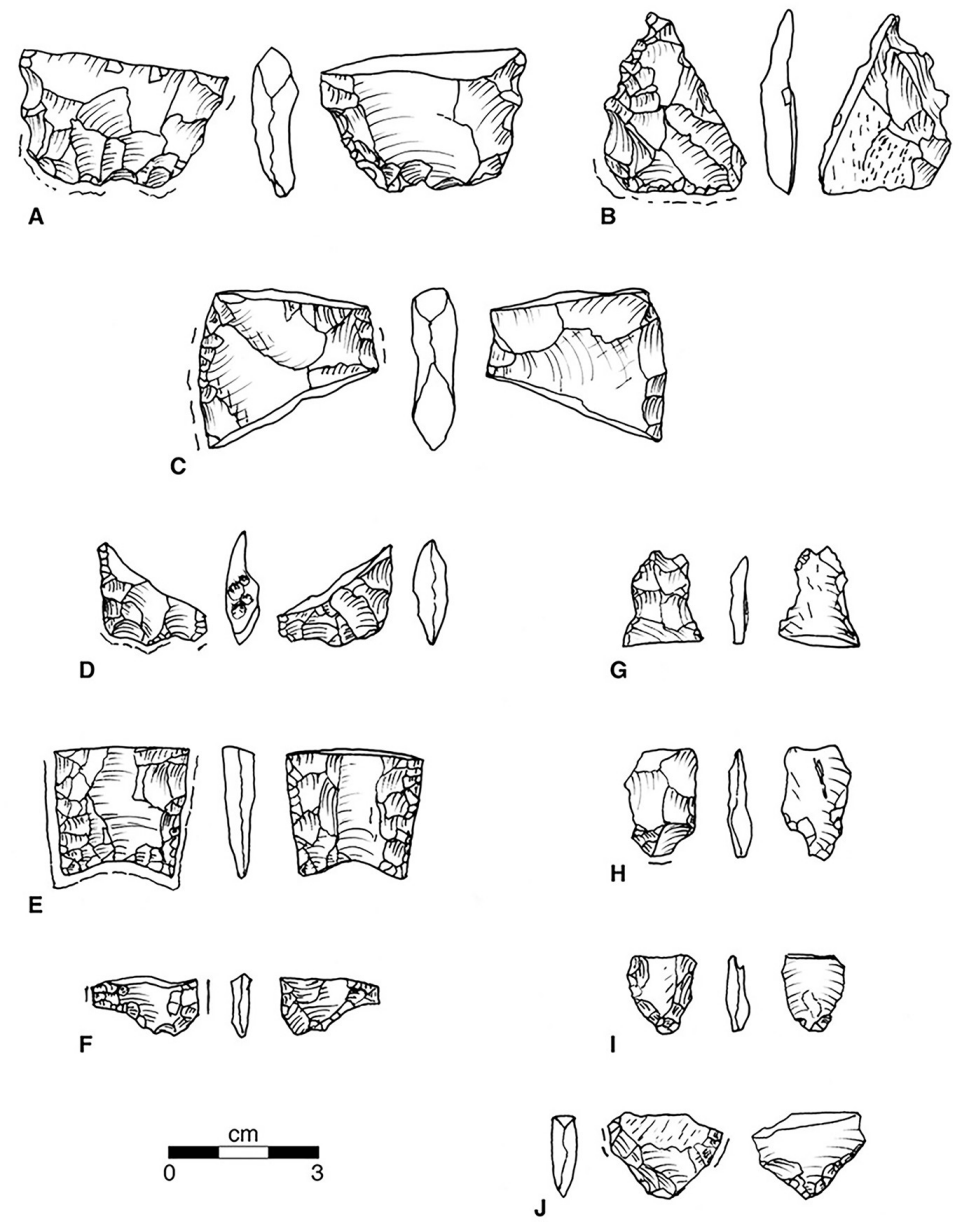
Bifaces and related items. (A) Base of Primary Biface. Belson N458/E493 Surface, Axial Ln: 2.68+, Max. Wd: 4.28, Max Thk:..92, Base-to-Max. Thk: 2.54, Weight: 13.6 gm. Medium Banded Green and White: Attica White/Green. (B) Base of Primary Biface with Denticulate retouch. Belson N463.5/ 501.0, Plow zone. Axial Ln: 3.33+, Max. Wd: 2.89, Max. Thk:..80, Base-to-Max. Thk: 1.54, Weight: 8.7gm, Fine Light Tan chert with gray and white mottling: Probable Liston Creek. (C) Midsection of Primary Biface. Belson N453/E516 Surface, Axial Ln: 3.31+, Max. Wd. 3.90, Max Thk:..96, Weight: 14.1 gm. Fine Banded Green and White chert: Attica White/Green. (D) Base of Secondar Biface. Belson N463.85/ E498.1, 99.44 AD. Axial Ln: 2.05+, Max. Wd: 2.35+, Base-to-Max Wd: 1.95+, Max. Thk:..73, Base-to-Max. Thk: ca 1.31+, 1st Flute Ln:..90, 1st Flute Wd:..65, No other flutes, Basal Concavity: -18. Weight: 2.5 gm, Fine Banded Gray and Brown chert: Paoli. (E) Base of Finished Fluted Point. Belson N466.9/ E498.1, 99.22–9947. AD. Axial Ln: 2.66+, Max. Wd: 2.81+, Max. Thk:..60, Base-to-Max. Thk: ca 2.70, 1st Flute Ln: 2.20+, 1st Flute Wd:..89, 2^nd^ Flute Ln: 2.10, 2nd Flute Width: 1.64, Inter flute Thk:..52, Basal Concavity:..46, Weight: 6.0gm, Heavy lateral grinding. Medium Mottled Blue and Gray chert: Attica Blue/Grey. (F) Midsection of Fluted Point. Belson N481/E532 Surface. Axial Ln:1.16+, Max. Wd. 2.15, Max Thk:..38, Inter flute Thk:..35, 1st Flute Wd: 1.05, 2nd Flute Wd:..95, Weight: 0.9 gm. Fine Blue and Gray chert: Attica Blue/Gray. (G) Distal end, Channel Flake. Belson N464.97/ E498.31, 99.35 m AD. Axial Ln: 2.03+, Max. Wd: 1.22+, Max. Thk:..33, Weight: 1.0 gm, Medium Banded Gray and Green chert: Attica White/Green. (H) Proximal end, Channel Flake: Belson N462/ E 498, plow zone. Axial Ln: 2.19+, Max. Wd: 1.50, Base-Max Wd: 1.76, Faceted Platform:..57x.23, Weight: 1.2 gm. Fine Banded Gray chert, Attica Blue/Gray. (I) Proximal end, Core-struck Blade. Belson N465-498, plowzone. Axial Ln: 1.72+, Max. Wd. 1.42, Base-to-Max Wd: 1.37, Max Thk:..31, Base-to-Max Thk: 1.46 Flat Platform:..46 x..42, 80° Angle, Weight..97 gm. Fine Blue and Gray chert: Attica Blue/Gray. (J) Proximal end, Core-struck Blade: Belson 485N/ 486E Surface, Axial Ln: 1.72+, Max. Wd: 2.48+, Base-to-Max Wd: 1.50+, Max Thk:..46, Base-to-Max Thk: 1.48+. Flat Platform..35 x..18, 30° Angle, Weight: 2.40 gm. Medium Blue and Gray chert, Attica Blue/Gray.

We now have a series of oval biface fragments that illustrate preforms brought from the sources (Fig 7A–7C). Initial short expanding marginal retouch creates the rounded, oval form without completely removing the surfaces of the original tabular piece or large flake (Fig 7A and 7B). The second of these was of Liston Creek chert, re-purposed as a denticulate tool. Additional overlapping marginal expanding thinning flakes cross over the mid-line creating a symmetrical lenticular primary biface (Fig 7C). Steep short edge flakes suggest this biface was used as a beveled knife, a tool known in several other Great Lakes fluted point assemblages [35: p.48-50 Fig 43, 30: Figs 6a and 7a, 34: Figs 6.4f, g). This suggestion requires testing with use-wear analysis. An informative link between such secondary bifaces (also termed “late stage preforms” by Waters and Jennings [55: p.38-71 Figs 17–61], and the fluted points for which they serve as preforms, is provided by the damaged base of a small biface (Fig 7D) of Paoli chert from north-central Kentucky. The convex base was ground, and a first successful basal thinning flake was struck on the right. The base was then refurbished and ground on the left, but efforts to remove a second basal thinning flake miscarried leading to an oblique fracture of the biface. We also have a fully finished and ground midsection (Fig 7F) and a fully finished and ground fluted point base (Fig 7E, excavated in sub-plow deposits). The proteomic evidence from the latter is discussed in the section, *Animal exploitation indicated by initial residue studies*. Two channel flake fragments, one complete and one proximal (Fig 7G and 7H), imply that secondary bifaces were actually fluted at Belson.

**Fig 8:**
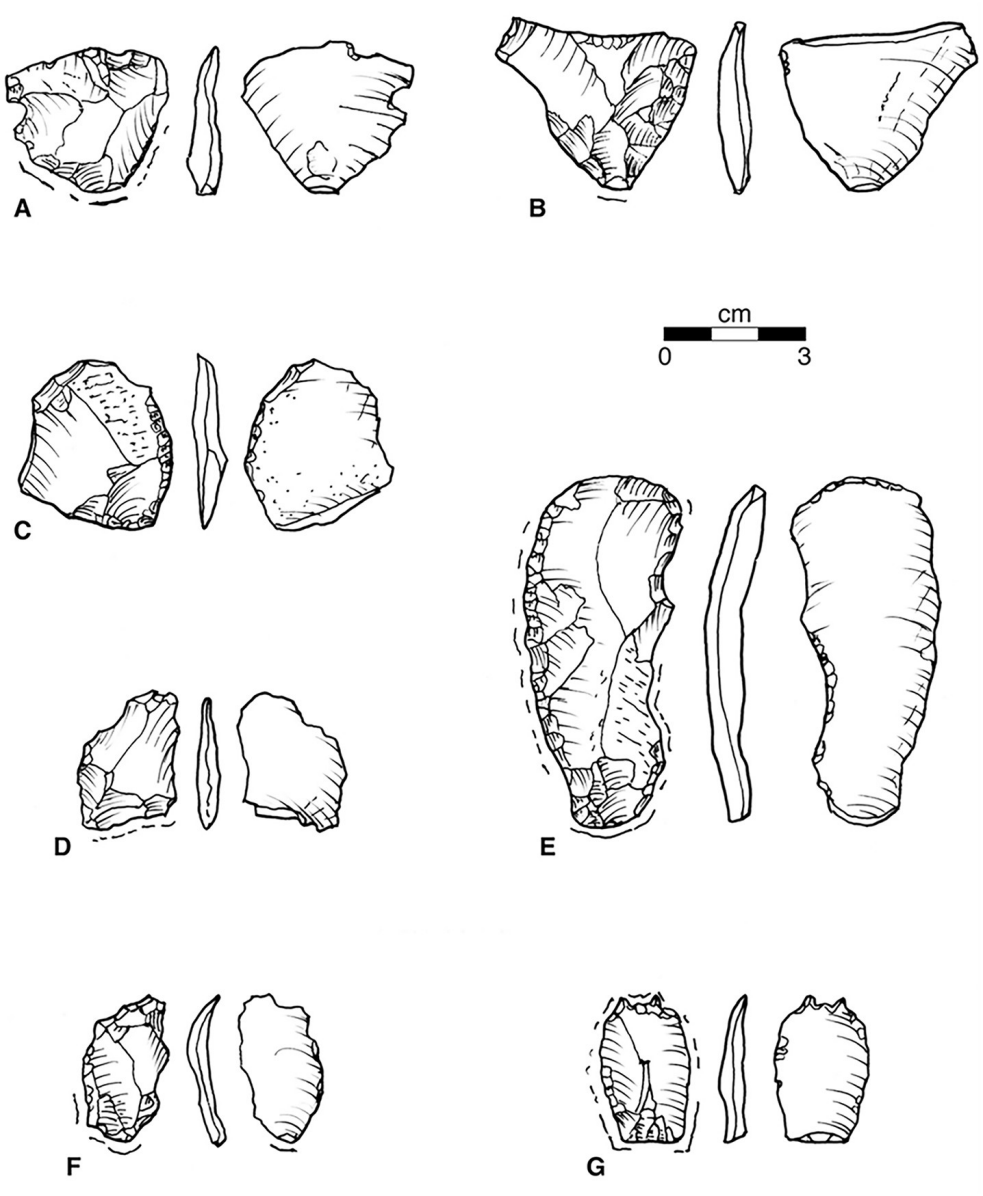
Flakes, Convex Edge Scrapers, and Gravers. (A) Biface Side-Thinning Flake with Notch. Belson N479/ E497 Surface. Axial Ln: 2.92, Max. Wd: 3.23, Max. Thk:..55, Base-Max thickness: 2.14, Base-to-Max Thk: 1.98, Ground, flat Platform..53 x..32, 85° Angle, Notch Wd:..58, Notch Depth:..34. Weight 4.5 gm. Fine Gray and Tan: Attica Gray\Tan. (B) Biface Side-Thinning Flake with Convex Edge Retouch. Belson N462.49/ 495.29, 99.32 m AD. Axial Ln: 3.55, Max. Wd: 4.15, Base-Max. Wd: 2.72, Max. Thk:..51, Base-to-Max. Thk: 1.56 Ground flat Platform..65 x..24, 80° Angle, Scraper Edge Ln: 3.15, Scraper Edge Depth: 1.25, Scraper Edge thickness:..28 Edge Angle:..20, Weight: 6.5 gm. Fine Banded Green and white chert: Attica White/Green. (C) Flake fragment with Convex Edge Retouch. Belson Surface. Axial Ln: 3.39+, Max. Wd: 3.11, Base-Max. Wd: 1.20, Max. Thk:..48, Base-to-Max Thk: 2.15, No platform, Scraper Edge Ln: 2.50, Scraper Edge Depth:..34, Scraper Edge Thk:..36, Edge Angle: 30°. Weight: 5.3 gm. Fine Banded Blue and Green chert: Attica Blue/Gray. [courtesy of Steve Zarza]. (D) Flake Fragment with Notches and possible broken-off gravers. Belson N463-E497, Plow zone. Axial Ln: 2.46+, Max. Wd: 2.07, Max. Thk:..39, No platform. Notch Wd:..50, Notch Depth:..17, Weight: 1.4 gm. Medium Banded Gray and Green chert: Attica: White\Green. (E) End-thinning Blade with Convex Edge Retouch. Belson N467.21/ E497.85, 99.45–99.50 m AD. Axial Ln: 6.61, Max. Wd: 2.96, Base-Max. Wd: 6.27, Max. Thk:..78, Base-to-Max. Thk: 3.90. Ground, Flat Platform 1.10? x..32, 30° Angle, Scraper Edge Ln: 5.28, Scraper Edge Depth:..33, Weight: 15.0 gm, Medium Banded Blue/Gray chert: Attica Blue/Grey. (F) Biface Side-Thinning Flake with possible broken-off Gravers. Belson N458/ E522 (2019 Test #1). Axial Ln: 2.84, Max. Wd: 1.64, Base-Max. Wd: 1.56, Max. Thk:..25, Base-to-Max. Thk: 2.27, Flat Platform..40 x..04?, 80° Angle, Weight: 1.2 gm. Fine Translucent Banded Tan chert: Possible Upper Mercer variant. (G) Biface Side-Thinning Flake with Double Gravers. Belson N458/ E497 Surface. Axial Ln: 2.87, Max. Wd: 1.80, Base-Max. Wd: 1.81, Max. Thk:..35, Base-to-Max. Thk: 1.46, Faceted Platform 1.25 x..36, 70° Angle, Weight: 1.2. Fine Banded Blue and Gray chert: Attica Blue/Gray.

The often expanding biface thinning flakes struck from the edges of large primary bifaces were systematically used to make several different kinds of tools. Some are notched with multiple flake removals from the same point using the inner flake surface as a platform. These often have tiny flakes from utilization on another edge (Fig 8A). We suspect these notched tools have a specific use, and hope that evidence of use-wear and residue analysis will resolve this problem. Others have a long, usually convex edge with regular unifacial retouch (Fig 8B and 8C) traditionally referred to as ‘side scrapers’. The retouch is variable in size and pattern, and we doubt they have one specific use. A long, narrow edge removal flake from a primary biface of Attica chert (Fig 8E) has a similar long convex edge with regular unifacial retouch on one edge, and, surprisingly, visible wear like an end-scraper on the distal end. Similar tools from other fluted point assemblages have been retouched to a point [45: Fig 5a, 56: p.351 Fig 6a, 57: Plate II 13, 15, 16, Plate III 3, 28, 30]. Finally, there are two similar small tools with graver tips, or remnants of snaped-off tips, though made from flakes of different derivation. One is a biface thinning flake struck across the face of a secondary biface from a ground platform (Fig 8F). The raw material is exceptional, a fine translucent tan and brown chert, perhaps from a source in East-Central Ohio. The other is a biface thinning flake of Attica with two broken off gravers (Fig 6D). Another appears to be from a blade struck from an Attica core with a flat platform at 80° to the line of strike. Here, the two gravers are well preserved (Fig 8G). There are also two proximal ends of blades with prepared blade-core platforms, at near 90° without further modification (Fig 7I and 7J).

**Fig 9:**
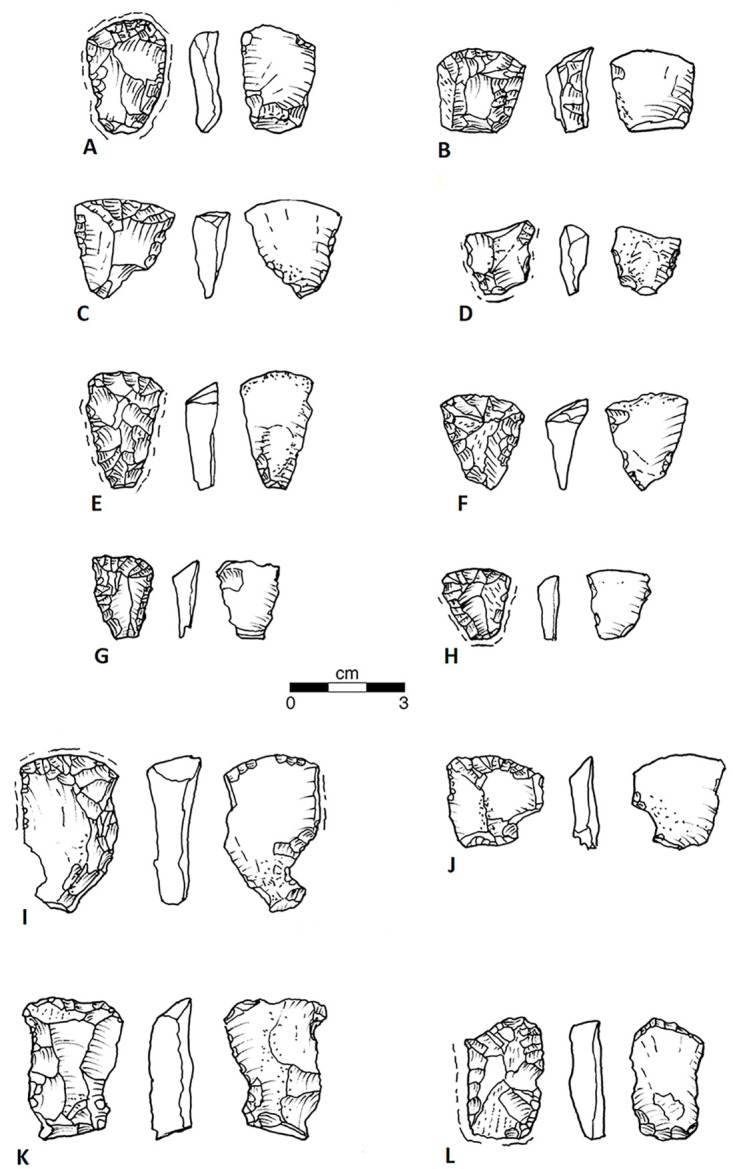
Small triangular end-scrapers, and larger rectangular end-scrapers. (A) Triangular End Scraper, lightly used. Belson Surface. Axial Ln: 2.99, Max. Wd: 2.14, Base-to-Max. Wd: 2.45, Max. Thk:..76, Base-to-Max Thk: 1.80, Ground flat Platform 1.16 x..62, 90° Angle, Bit Wd: 2.15, Bit Depth:..49, Bit Thk:..72, Bit Angles: 35°/70°, Weight: 5.3 gm. Fine Banded Green and White chert: Attica White/Green. (B) Triangular End Scraper, lightly used. Belson N464/ E500 Plow zone. Axial Ln: 2.37+, Max. Wd: 2.47, Base-to-Max. Wd: 1.80, Max. Thk: 1.09, Base-to-Max Thk: 1.69, Ground flat? Platform? Bit Wd: 2.47, Bit Depth:..44, Bit Thk: 1.09, Bit Angle: 50°, Weight: 7.9 gm. Fine Banded Gray and White chert: Attica Blue/Gray. (C) Triangular End Scraper, lightly used Belson Surface. Axial Ln: 2.82, Max. Wd: 2.69, Base-to-Max. Wd: 2.35+, Max. Thk:..91, Base-to-Max Thk: 1.95+, No Platform, Bit Wd: 2.69, Bit Depth:..30, Bit Max. Thk:..95, Bit Angles: 65°/ 80° Weight: 6.0 gm, Fine Mottled Gray chert: Constantine. (D) Butt of Triangular End Scraper, lightly used. Belson N461.49/ E496.60, 99.45 AD. Axial Ln: 1.60+, Max. Wd: 1.97. Base-to-Max. Wd: 1.65, Max. Thk: 65, Base-to-Max. Thk: 1.60, Ground flat Platform:..88 x..30, Angle 80°, Weight: 2.4 gm. Medium Banded Green and Gray: Attica White/Green. (E) Triangular End Scraper, reworked and used. Belson N463 / E528 Surface. Axial Ln: 3.28, Max. Wd: 1.95, Base-to-Max. Wd: 2.92, Max. Thk:..79, Base-to-Max Thk: 2.71, Ground flat Platform..68 x..34, Angle 80°, Bit Wd: 1.91, Bit Depth:..35, Bit Thk:..87, Bit Angles: 40°/70°, Weight: 6.0 gm, Fine Banded Gray and Green chert: Attica White/Green. (F) Triangular End Scraper, reworked and used. Belson N463/E498 Surface. Axial Ln: 2.74, Max. Wd: 2.41, Base-to-Max. Wd: 2.43, Max. Thk:..92, Base-to-Max Thk: 2.03, No Platform, Bit Wd: 2.37, Bit Depth:..35, Bit Thk:..68, Bit Angle: 45°, Weight: 4.5 gm. Fine Banded Gray and Green chert: Attica White/Green. (G) Triangular End Scraper, reworked and used. Belson N473/ E493 Surface. Axial Ln: 2.28+, Max. Wd: 1.83, Base-to-Max. Wd: 2.05, Max. Thk:..60, Base-to-Max Thk: 1.82, No Platform, Bit Wd: 1.83, Bit Depth:..23, Bit Thk:..60, Bit Angles: 30°/70, Weight: 2.5 gm. Medium Banded Gray and Tan chert: Attica Blue/Gray. (H) Triangular End Scraper, reworked and used. Belson N464/ E497, Plow zone. Axial Ln: 1.98, Max. Wd: 1.76, Base-to-Max. Wd: 1.73, Max. Thk:..49, Base-to-Max Thk: 1.55, No Platform, but retouched base ground, Bit Wd: 1.76, Bit Depth:..15, Bit Thk:..49, Bit Angles: 40°/60°, Weight: 1.9 gm. Fine Banded Blue and Gray chert: Attica Blue/Gray. (I) Rectangular End Scraper with edge notch, finished, little used. Belson N457 E570 Surface. Axial Ln: 4.28, Max. Wd: 2.66, Base-Max. Wd: 3.68, Max. Thk: 1.39, Base-to-Max. Thk: 3.51. Platform? Bit Wd: 2.66, Bit Depth;..53, Bit Thk: 1.39, Bit Angles: 60°/ 90°, Weight: 14.4 gm. Medium Banded White and Green chert: Attica White/Green. (J) Rectangular End Scraper with edge notch, finished, much used. Belson Surface. Axial Ln: 2.53+, Max. Wd: 2.60, Base-Max. Wd: 2.25, Max. Thk:..60, Base-to-Max. Thk: 1.88. No Platform, Bit Wd: 2.56, Bit Depth:..35, Bit Thk:..60, Bit Angle: 50°, Weight: 4.8 gm. Fine Banded Blue and Green chert: Attica Blue/Gray. (K) Rectangular End Scraper, unfinished, little used. Belson E497 N460 Surface Axial Ln: 3.85, Max. Wd: 2.70, Base-Max. Wd: 3.58, Max. Thk: 1.18, Base-to-Max. Thk: 2.14 No platform. Bit Wd: 2.70, Bit Depth:..33, Bit Thk: 1.18, Bit Angles: 28°/?°, Weight: 13.0 gm. Medium Gray and Tan chert: Attica Gray/Tan. (L) Rectangular End Scraper, much used. Belson N473/ E488 Surface. Axial Ln: 3.21, Max. Wd: 2.04, Base-Max. Wd: 1.50, Max. Thk:..92, Base-to-Max. Thk: 1.65, Faceted Platform. 1.39 x..38, Angle: 95°. Bit Wd: 2.01, Bit Depth:..10, Bit. Thk:..77, Bit Angle: 95°, Weight: 6.8 gm. Medium Banded Blue and Gray chert: Attica Blue/Gray.

As of the end of the 2021 season the most common kind of tool in the Belson assemblage is the end scraper, distinguished by a convex working edge or ‘bit’ with roughly convergent lamellar end-flaking. Many show visible polish on both the inner and outer faces and some had irregular crushing on both the left and right edge, which we argue is from being used while fitted into a socket. These could be re-sharpened in the handle, shortening the scraper along its axis and steepening and reducing the curvature or depth of the bit. However, we now realize this is only one step in the process. When the bit is re-sharpened such that it is almost flat, it must be removed from its handle and re-worked into a convex shape with low angle retouch, then re-fitted back into a smaller socket. This process was repeated until it could not be mounted in a handle. Among the end scrapers, there is a group of larger scrapers with roughly rectangular shapes (Fig 9I–9L). Initially, we thought these might be used hand-held without a handle, but most have the surface polishing we attribute to socketing (Fig 9I–9K). One is on a rough flake with little end retouch (Fig 9K), such that one might think it was a scraper preform, but it appears to have use-polish. Two have large notches in a proximal corner (Fig 9I and 9L). We do not know if this was a modification to fit the scraper into a handle or use damage caused by pressure against an ill-fitted handle. The remainder of the scrapers are smaller with a roughly triangular shape (Fig 9A–9H). Only one of these (Fig 9C) is made on a local raw material. All surviving platforms (Fig 9A, 9D and 9E) are flat and are at the proximal end of the scraper; all of these scrapers become wider and thicken toward the distal end of the tool. All the roughly triangular small scrapers with minimal edge modifications (Fig 9A, 9C, 9D, 9G and 9H) have triangular cross sections across the width, and the exterior flake facets are sometimes the shear-fractured and weathered surface of the original tabular piece of chert (Fig 9B, 9F and 9H). The easiest way to create such a flake would be to strike down on the corner of a tabular core. This proposition is best evaluated with debris from Late Glacial quarry workshops near the Attica source.

### Technological organization

**Fig 10:**
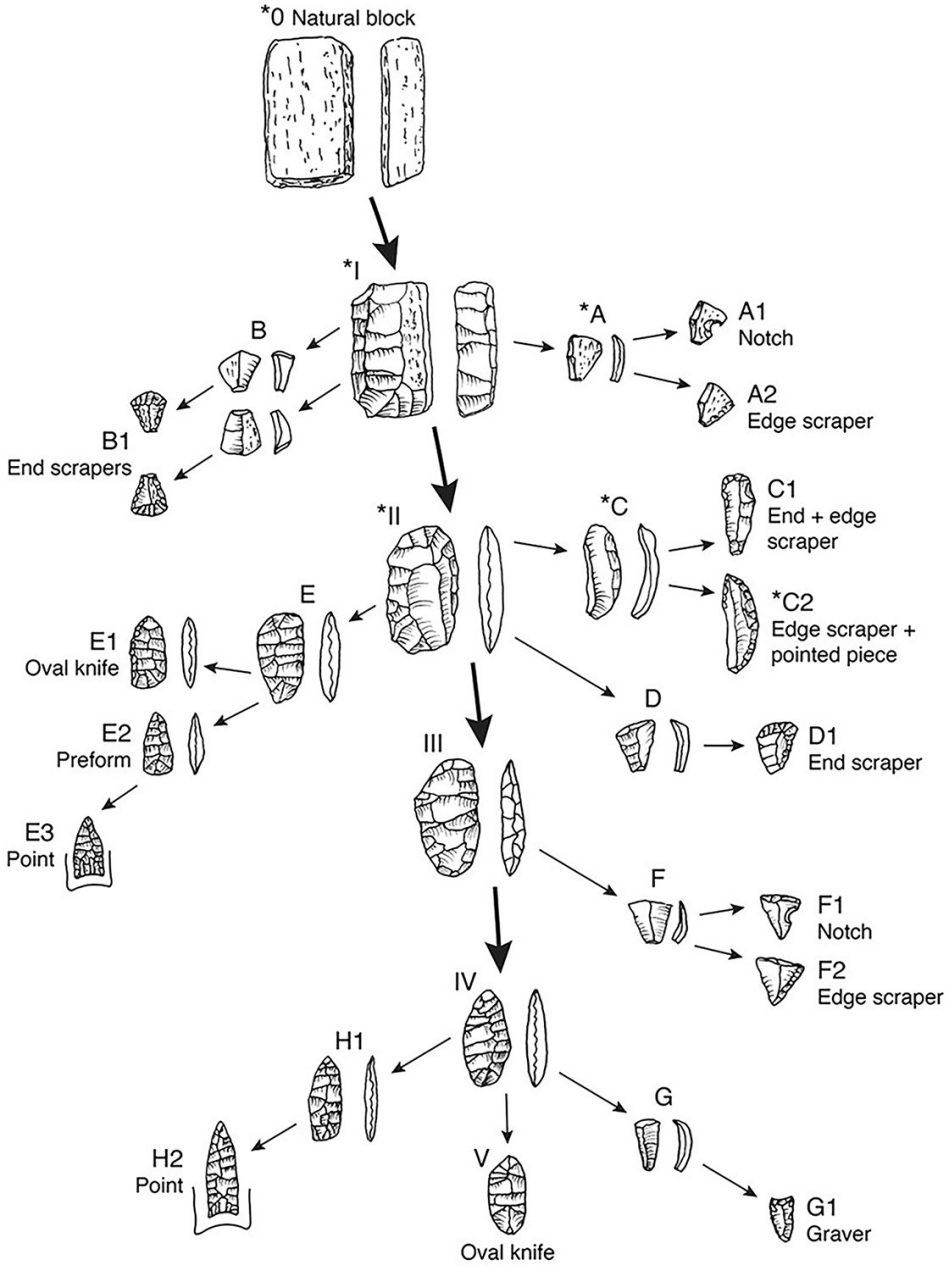
Industrial diagram for the Belson Clovis assemblage on Attica chert block and biface cores.

We can assemble the various linkages inferred above into an industrial sequence for Attica chert items, extending from the initial natural tabular piece to the finished tools. It is not a full ‘operational chain’ (eg. *chaine opérètoire*) or ‘industrial sequence’ because it does not include either the production of blade cores and striking of blades tools (because we do not yet have enough items from this trajectory to define it) or the re-sharpening of tools and the re-working of broken tools. Phases in the reduction of the original natural piece to core, primary biface, and secondary biface are denoted with Roman Numerals in the diagram (Fig 10). Steps in the modification of each kind of piece struck from these are indicated by capital letters A to H in square brackets. Note that these “steps” in the diagram simplify a continuous sequence of modifications. In the discussion below, references to specific tools follow in parentheses. Retouching of these flakes to make different tools are indicated by a letter and an Arabic number. This is a preliminary construct, one in which many steps in the sequence are represented by only one item in the recovered assemblage and some steps are conjectural (indicated by an asterisk). It serves as hypothesized network, one to be evaluated with the artifacts of Attica chert recovered during the next seasons of fieldwork at Belson.

The sequence begins with tabular blocks of chert (Fig 10 *0) selected from the source [49: p.15]. We have found no such tabular blocks at the Belson site, not surprising as the site is 235 km from the source and most tabular pieces must have been reduced to various kinds of cores and flakes. The early-stage flakes, however, inform us about the shape of the initial tabular piece. Flakes struck across the face of a tabular piece were expanding or rectangular (Fig 10 *A). The exterior face of a flake would have remnant geologically weathered surfaces. These could be used as is, or notched (Fig 10 A1), or given a linear retouch to form a scraper (Fig 10 A2). Some early removals were from the corners of tabular pieces. Flakes struck from a corner expand and thicken away from the platform (Fig 10B), with a weathered surface of the block often visible on one or more facets of the flake (Fig 9B, 9F and 9H). The distal ends of such expanding flakes could be given a steep convergent retouch to form a small end-scraper (Fig 10 B1). The recognition of the use of corner-struck flakes as preforms for small end-scrapers resolves an enduring conundrum in the study of Late Glacial industries. In his classic study of tools from the Shoop site, John Witthoft [57] realized many Shoop end-scrapers were made from expanding flakes but proposed these had been struck from the tip of a cone-shaped core, a physical impossibility. Subsequent analysts had no other solution. The effect of repeated removals from corners and faces would be to create a roughly oval core (Fig 10 *II). We have found no such cores at Belson, but Amick et al. [58] have reported these from the Tangier Cache near Attica and we expect to find fragments at Belson.

As the core was reduced, it became a primary biface, worked by distinctly different techniques of side-thinning, end-thinning and edge-thinning. Side-thinning flakes are very much like those struck across the faces of tabular pieces (Fig 10 IA) or secondary bifaces (which usually lack traces of weathered surfaces) and they were similarly retouched as scrapers and notches; they will not be discussed here. End- and edge-thinning flakes, however, were used to make some of the most distinctive tools found in the Belson assemblage and merit notice. On the outer surface they can exhibit the scars of both side-struck and previous end-struck flakes. These can be blade-like flakes curved along the edge of the biface (Fig 10 IIC) and have multiple forms of edge-retouch and notching (Fig 10 C1, Fig 8E) or pointed ends (Fig 10 *C2) well-known from related sites [45: Fig 5a]. Or they can be well-centered, either shorter (Fig 10D) and used for a large rectangular end scraper (Fig 10 D1) [cf. 23: Fig 2f], or longer and larger (Fig 10E), either backed and resharpened for use as an oval bifacial knife (Fig 10 E1, Fig 7C) [cf. 35: p.48-52 Figs 26, 43, 44], or further thinned (Fig 10 E2), fluted and ground for use as a projectile point (Fig 10 E3, Fig 7F). These should be smaller, thinner and more plano-convex than points made on later stage preforms (See Fig 10 H1, H2).

As the primary biface was reduced, becoming a secondary biface (Fig 10 III), usually by the striking of side-thinning flakes, typically expanding over-face flakes (Fig 10 F) which could be notched (Fig 10 F1, Fig 8A) or retouched as a scraper (Fig 10, Fig 8B). The remnant smaller advanced secondary biface (Fig 10 IV) could be further reduced by similar expanding flakes or narrow blade-like flakes (Fig 8F) which could be modified with two or more graver spurs (Fig 10 G1, Fig 8G) [cf. 23: Fig 2e]. The advanced secondary biface could be given a beveled, ground convex base (Fig 10H), from which multiple flutes could be struck (Fig 7D) creating slightly concave base, then laterally ground (Fig 10 H1) [cf. 23: Fig 5b] for mounting on a handle probably for use as knife point [23: Fig 2a] or for mounting on a shaft probably for use as a projectile point (Fig 7E). The advanced secondary biface could also be used with little further modification as an oval knife (Fig 10V). Points and knives made on secondary bifaces should be larger, thicker, and more bi-convex in section than points made on other preforms (Fig 10 E2, E3).

In sum, the technological organization indicated by the Belson flaked stone industrial sequence for the industry on Attica chert is well-structured. However, examination of Fig 8 shows that at Belson there was tactical flexibility, no doubt to optimize a diminishing stock of chert from far-away, with at least three pathways ways to produce a heavy edge scraper (Fig 10 A2, C2, F2), three pathways ways to produce a smaller end scraper (Fig 10 B1(2), D1) and two pathways ways to produce a fluted point (Fig 10 E3, H2). Though different in detail from the Attica industrial sequence for the contemporary Palmer site [44: Fig 7] the number of options are similar. Other Eastern sites are reported to have more structured industrial sequences with each finished tool usually made from one type of flake blank [e.g., 57]. This, however, may result from a traditional categorical emphasis on ‘tool types’. When every flaked stone product is represented in the industrial sequence, as was first demonstrated in the careful analysis of Michael Shott [34] of the Leavitt Site, a later fluted point assemblage from central Michigan, the tools manifest the same tactical flexibility noted at Belson and Palmer.

## Animal exploitation indicated by initial residue study

Results from identification of proteins from three lithic artifacts recovered from the Belson Site are reported by Scott Cummings and Maison [59]. See supplementary information for a summary of the results (S11 Fig). The protein residue analysis for lithic artifacts was performed by the PaleoResearch Institute in Colorado using counter immune-electrophoresis (CIEP) [60,61] as modified by Newman and Julig [62] and PaleoResearch for archaeological samples. See supplemental material for details. A detailed description of materials and methods can be found in the S2 Appendix.

The criteria for submission to *PaleoResearch* was that the item be observed *in situ* below plow disturbance, that it not been touched by human hands, and that there was both sediment attached to the item and sediment near the item to serve as a control. There are other small flakes from the 2020 and 2021 season that fit these criteria, but the authors chose to submit three formal tools: one finished point base (Fig 7 E), one multi-use tool on an end-thinning blade (Fig 8E), and one scraper on a biface thinning flake (Fig 8B). The others will be studied when funds are available. These analyses demonstrate the potential of proteomic methods for chert artifacts in Late Glacial contexts such as the Belson Site.

### Results of the residue study

The one finished Fluted Point base found below the plowzone (Fig 7E) (Artifact 1) has a very weak positive indication of suid proteins, which most parsimoniously indicates the presence of tayassuid proteins from the closely related *Platygonus* peccary (both the suidae and tayasuidae form the superfamily suoidea), and very weak positive indications of the proteins of lagomorphs, most likely a rabbit (*Sylvilagus)* or a hare (*Lepus)* in this area. There is no indication of suid or lagomorph proteins in the control samples. See supplemental information of a photograph of the excavated fluted point base (S12 Fig).

A Lateral Edge-thinning Flake, probably from a Primary biface, with Convex Edge Retouch (Fig 8B) (Artifact 2) has weak positive indications of the proteins of a cervid which could have been deer (*Odocoileus*), caribou (*Rangifer*), elk (*Cervus*), or moose (*Alces*) in this area. There is no indication of cervid proteins in the control samples.

An End-thinning Blade with Convex Edge Retouch (Fig 8E) (Artifact 3) has very weak probable positive indication of the proteins of proboscideans which were not considered conclusive. There is also a very weak positive of the protein of a bovid, which in the Late Glacial midcontinent could have been either musk ox (*Ovibos*) or bison (*Bison*). There is no indication of proteins of proboscideans or bovids in the control samples. There were also indications of proteins from canids and equids, but these proteins were also found in nearby control samples and since domestic horses were used to cultivate these field during the19th and 20^th^ centuries and domestic dogs and wild foxes still range over the area, we cannot reject the hypotheses that these proteins were from recent feces or other tissues.

### Conclusion for the residue study

In sum, there are indications for the butchering of sheep/musk ox and cervids on two retouched flakes, and of smaller mammals on a point base. These are indicative of more opportunistic foraging rather than specialized “big game” hunting. This said, if any of the larger herbivores were systematically hunted and their products were regularly stored [63] their contribution to food resources would greatly outweigh those of smaller animals. Clearly more proteomic study of stone tools is warranted, however see Gingerich [64].

## Summary and conclusions

The First two seasons of excavation at the Belson Site have yielded evidence relevant to a range of theoretical issues concerning foraging behavior. This was greatly facilitated by having a substantial lithic assemblage, and evidence for multiple occupations largely undisturbed below modern ploughing. The technological organization understood from the Belson material supports existing models for Clovis assemblage structure including the use of triangular scrapers, modified flakes and blades, and fluted bifaces created with aggressive, over-face flaking and early fluting [14,13]. Having a buried component also allowed us to begin a proteomic study aimed at testing models for animal exploitation. Initial results suggest that a broad-spectrum foraging model considering the specifics of the local environment—as modeled by Cannon and Meltzer [15]—would be appropriate for characterizing subsistence pattern at the Belson, rather than traditional models that describe Clovis foragers as specialized large mammal hunters.

In terms of foraging and mobility models, the chert sourcing data from Belson seems congruent with an ‘outcrop centered’ model where high-quality chert outcrops serve as central places on the landscape coalescing of macro bands [24,26]. The Belson site is situated on a river terrace with a good view to the south, over a river channel that herds of herbivores would cross in the Spring. The site likely served as a relatively short-term camp where people, who went northwest from the Attica source in order to position themselves to take advantage of migrating animals, while hunting smaller and more varied game opportunistically, as well as engaging a spectrum of technology maintenance. Moreover, we have evidence of multiple visits to the site with the layout of activities being similar for successive visits. This organization of standardized tasks seems to suggest a fixed strategy in the dispositions of logistical tasks, supporting Binford’s [53,54] model for foraging in a new environment. Additional excavations at the site will provide data to refine our interpretations from the first two seasons and begin to start making site-specific foraging models.

## Supporting information

S1 Fig Drone photograph from season 1 in 2020 looking northwest. Intended to present the excavation as it appeared in the field near the end of the excavation seasons, and to give readers a visual understanding of the site and excavation context. (Photo credit Tommy Talbot).
(TIF)

S2 Fig Drone photograph from season 1 in 2020 looking down. The photograph is oriented south up. Intended to present the excavation as it appeared in the field near the end of the excavation seasons, and to give readers a better visual understanding of the site and excavation context. (Photo credit Tommy Talbot).
(TIF)

S3 Fig Belson central cluster excavation in September 2020. The initial N-S and E-W trenches are filled (north up). Feature 1 is cut by shadowed black square southwest of their juncture (Photo Credit Tommy Talbot).
(TIF)

S4 Fig Drone photograph from season 2 in 2021 looking down. The photograph is oriented with North to the right. Intended to present the excavation as it appeared in the field, and to give readers a visual understanding of the site and excavation context. (Photo credit Tommy Talbot).
(TIF)

S5 Fig Unit 102 before bisection of Feature 1. Show unit 102 after removal of the plow zone and first 10 cm of overlying sediment, but before bisection. Also, Dr. Wrights legs and scarf.
(TIF)

S6 Fig Image of the north face of Unit 102. Shows the north face of unit 102 before bisection to show Feature 1 in profile. Note that the profile is mostly loam without other pedogenic features. This will change after bisection.
(TIF)

S7 Fig Image of the north face of Unit 102 after bisection. This image shows unit 102 after bisection in the east-west direction to show Feature 1 in profile. Note the pedogenic features including clay patches, and iron oxide accumulations.
(TIF)

S8 Fig Additional photograph of unit 102 bisected to show Feature 1 in profile. This image shows more accurate color of sediments and soil features associated with Feature 1, and a micromorphology sample in place before being removed. Note the difference between the soil texture and features after bisection of the unit.
(TIF)

S9 Fig Size sorting diagram. This diagram shows flake elevations with black dots and a blue trend line showing the relative changes in size (weight) with depth. It is important to note that the cultural deposit is not at a consistent elevation, and that a majority of the flakes below 99.35 are from the bottom portions of Features 1 and 2.
(TIF)

S10 Fig Kernel density map of heated flakes. Map of heated flakes (teal dots), and non-heated flakes (open dots) over top of the kernel density map. Although it appears that the heated flakes concentrate in the feature areas, the percentage of flakes within and outside those areas is similar at just under 10%. Intended to show that there is an almost an even percentage of burnt flakes within Feature 1 as the rest of the excavation.
(TIF)

S11 Fig Summary of results table from the Paleo Research lab in Colorado. (TIF)

S12 Fig Image of both sides of the excavated Attica fluted point base 7E. (TIF)

S1 Appendix A series of three chi-square tests aimed at examining the significance of the difference in chert variants between the two occupations, and the difference between the plowed and the below plow deposit. (XLSX)

S2 Appendix Material and methods for the protein analysis from the PaleoResearch Lab. (DOCX)

S1 Dataset All the data from the flakes recovered during the Belson site excavations in 2020 and 2021. (XLSX)

S1 Text (DOCX)
