# Supplementary figures and images for: Clovis organizational dynamics at a Late Glacial campsite in the central Great Lakes: Belson site excavations 2020–2021
Source: PLoS One. 2024 May 29;19(5):e0302255. doi: 10.1371/journal.pone.0302255 (PMC11135731; doi:10.1371/journal.pone.0302255)

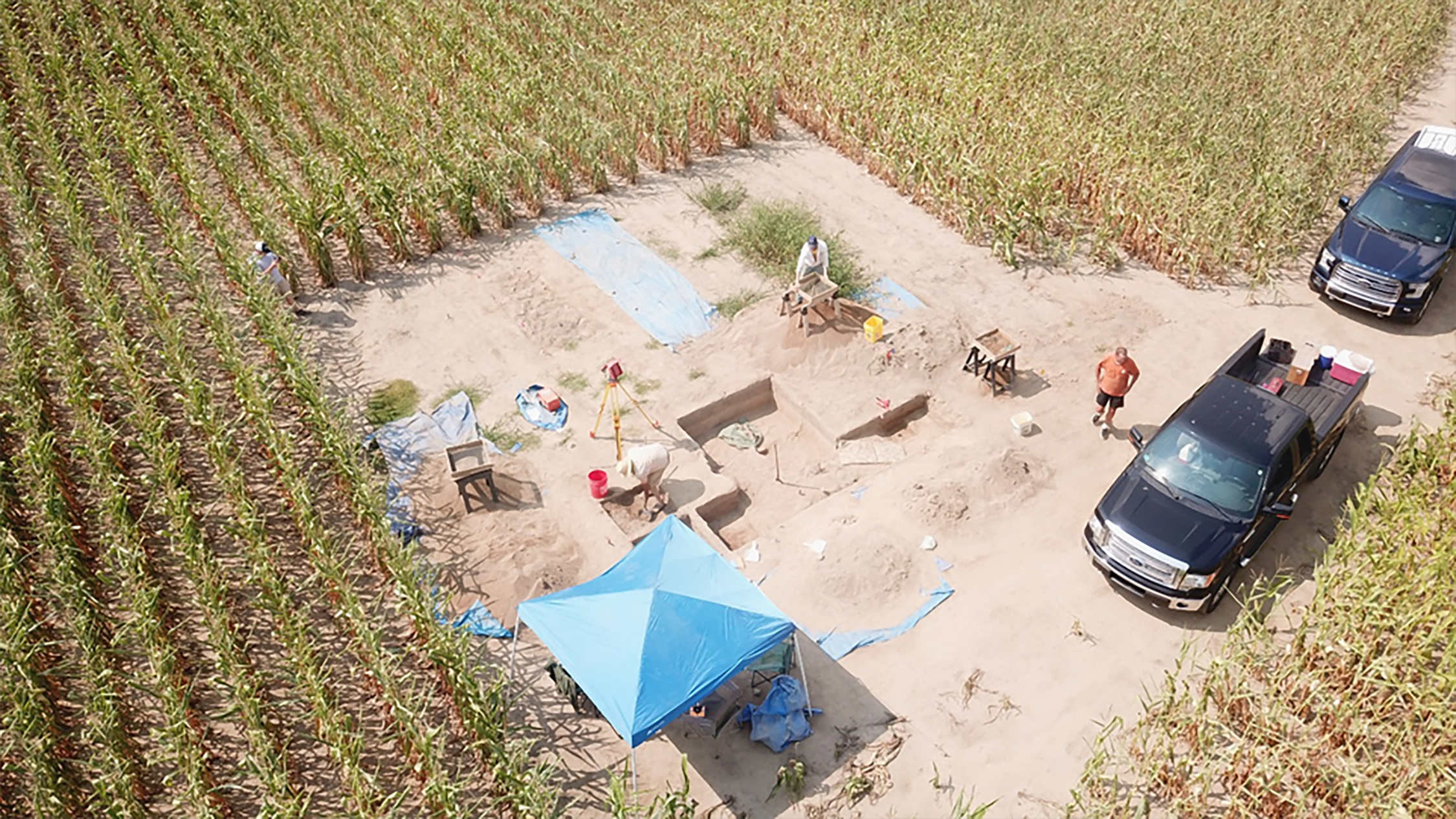

Supplement: S1 Fig — Intended to present the excavation as it appeared in the field near the end of the excavation seasons, and to give readers a visual understanding of the site and excavation context. (Photo credit Tommy Talbot). (TIF) [file pone.0302255.s001.tif]

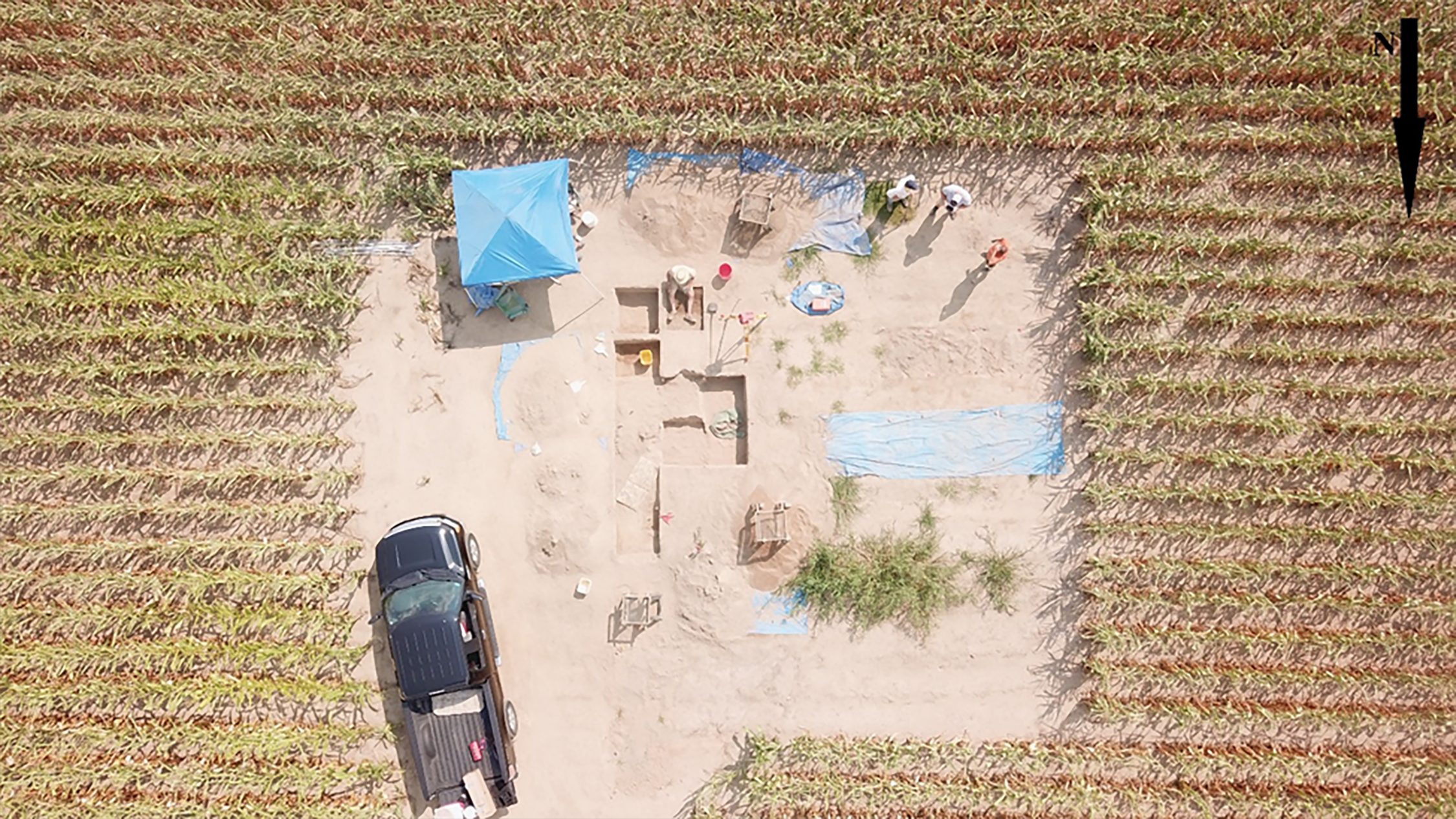

Supplement: S2 Fig — The photograph is oriented south up. Intended to present the excavation as it appeared in the field near the end of the excavation seasons, and to give readers a better visual understanding of the site and excavation context. (Photo credit Tommy Talbot). (TIF) [file pone.0302255.s002.tif]

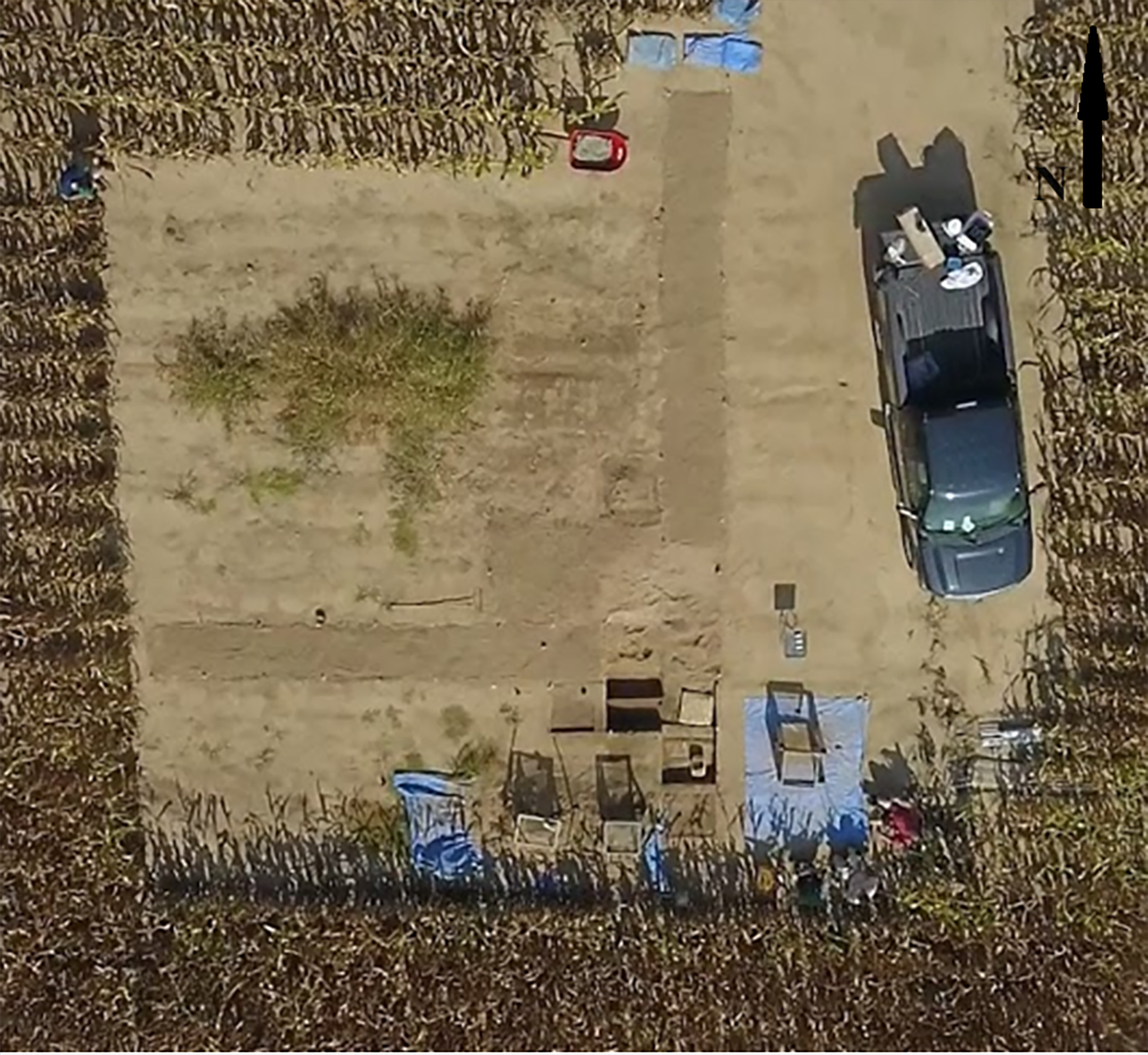

Supplement: S3 Fig — The initial N-S and E-W trenches are filled (north up). Feature 1 is cut by shadowed black square southwest of their juncture (Photo Credit Tommy Talbot). (TIF) [file pone.0302255.s003.tif]

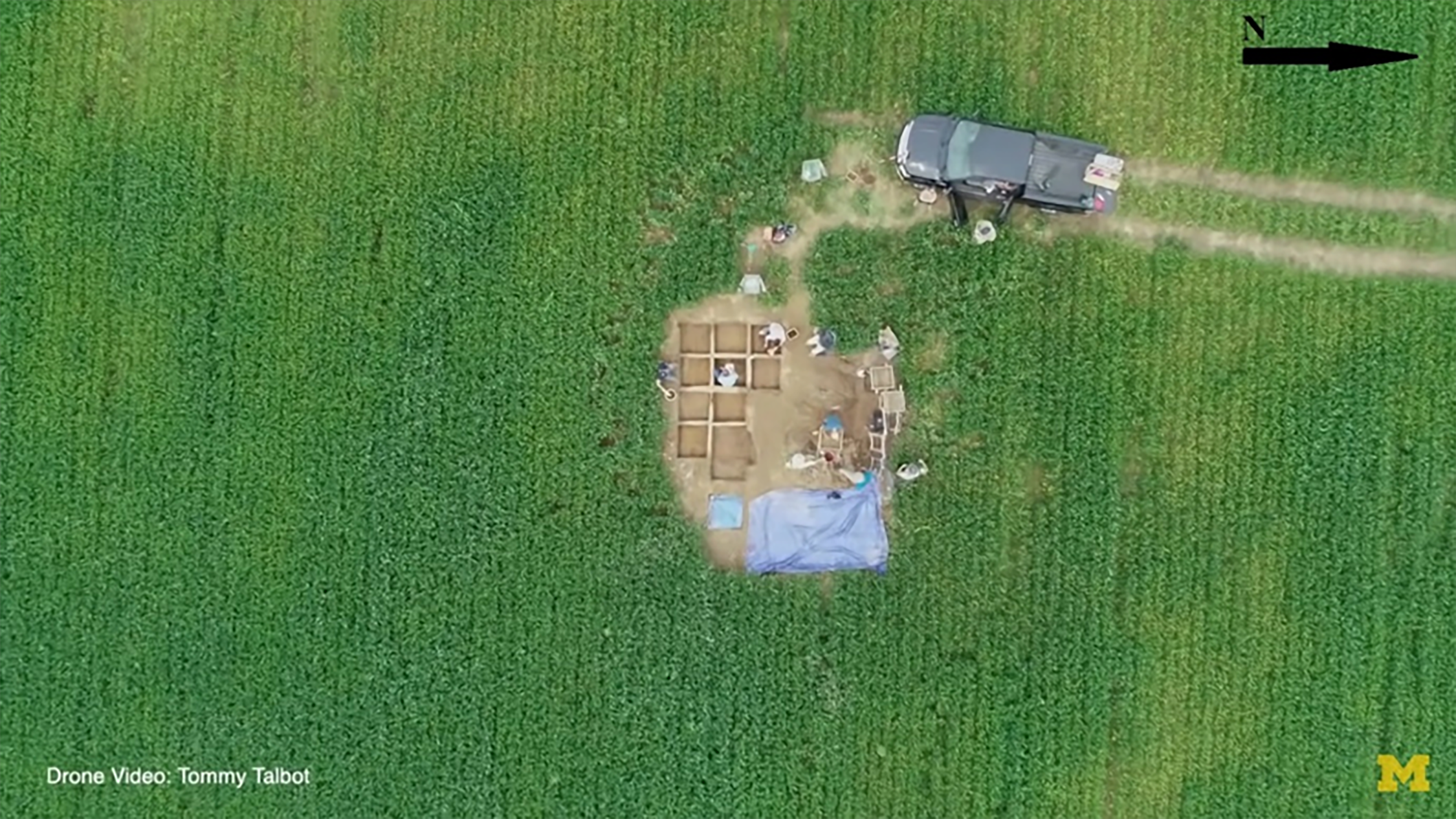

Supplement: S4 Fig — The photograph is oriented with North to the right. Intended to present the excavation as it appeared in the field, and to give readers a visual understanding of the site and excavation context. (Photo credit Tommy Talbot). (TIF) [file pone.0302255.s004.tif]

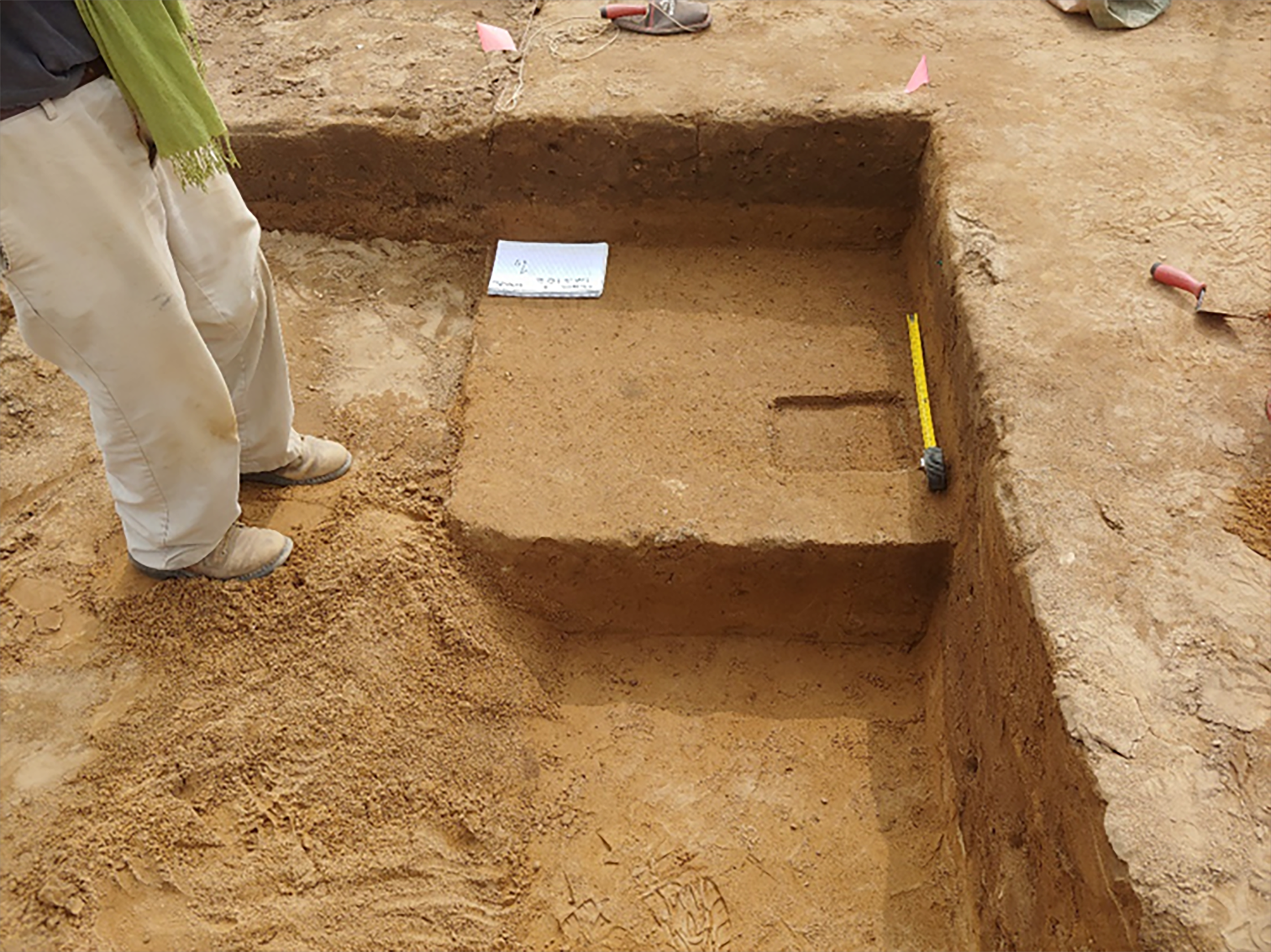

Supplement: S5 Fig — Show unit 102 after removal of the plow zone and first 10 cm of overlying sediment, but before bisection. Also, Dr. Wrights legs and scarf. (TIF) [file pone.0302255.s005.tif]

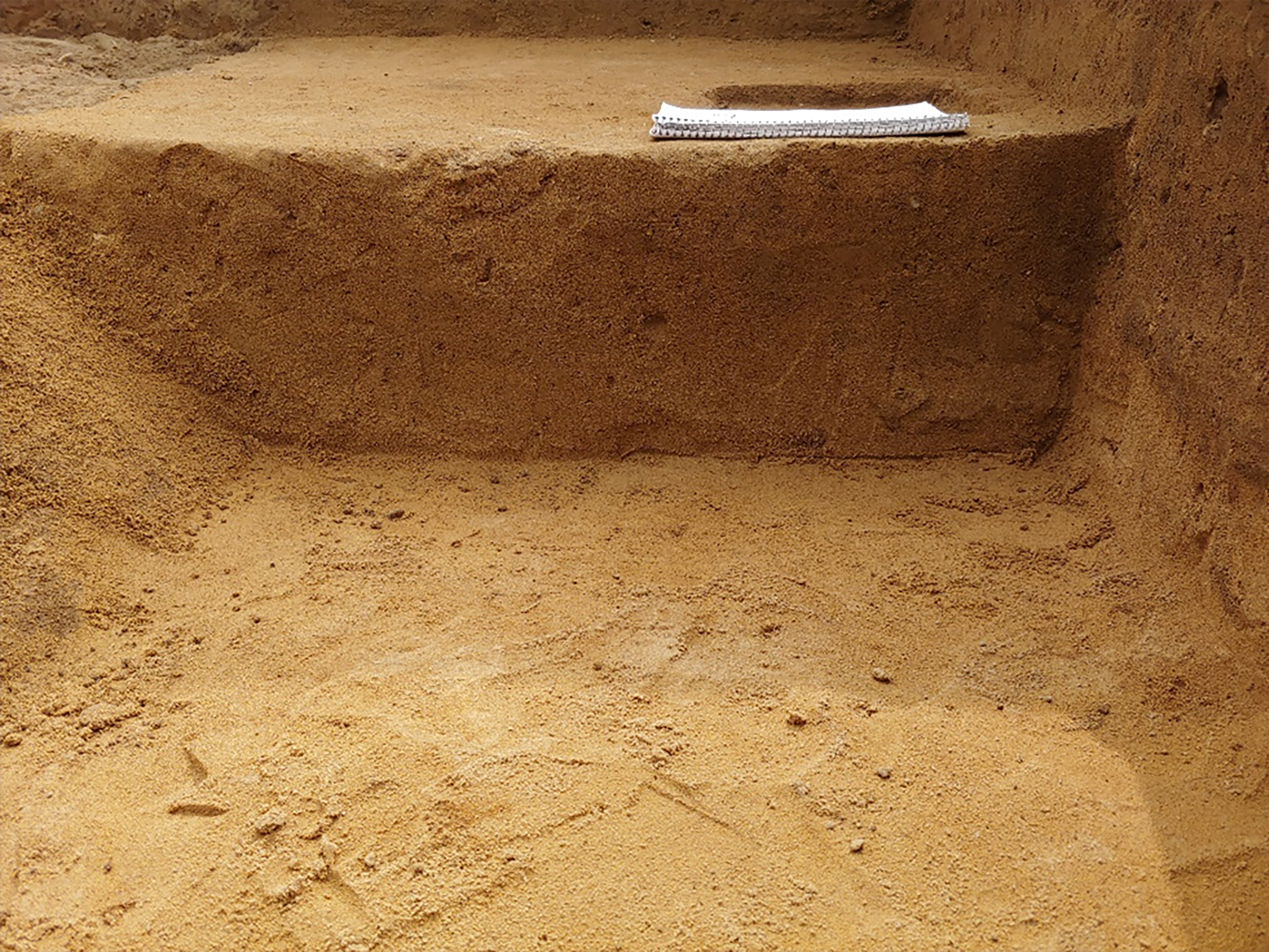

Supplement: S6 Fig — Shows the north face of unit 102 before bisection to show Feature 1 in profile. Note that the profile is mostly loam without other pedogenic features. This will change after bisection. (TIF) [file pone.0302255.s006.tif]

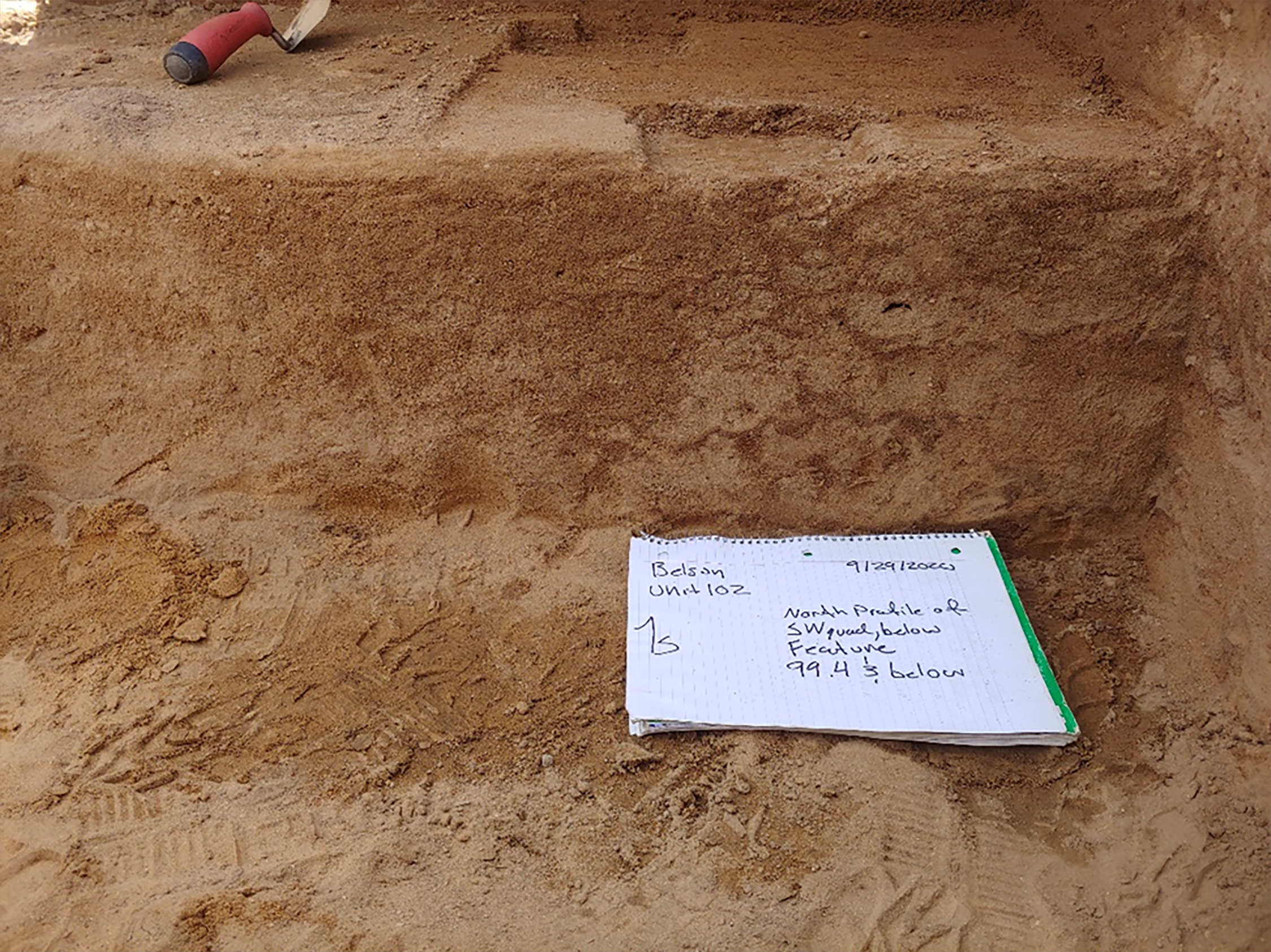

Supplement: S7 Fig — This image shows unit 102 after bisection in the east-west direction to show Feature 1 in profile. Note the pedogenic features including clay patches, and iron oxide accumulations. (TIF) [file pone.0302255.s007.tif]

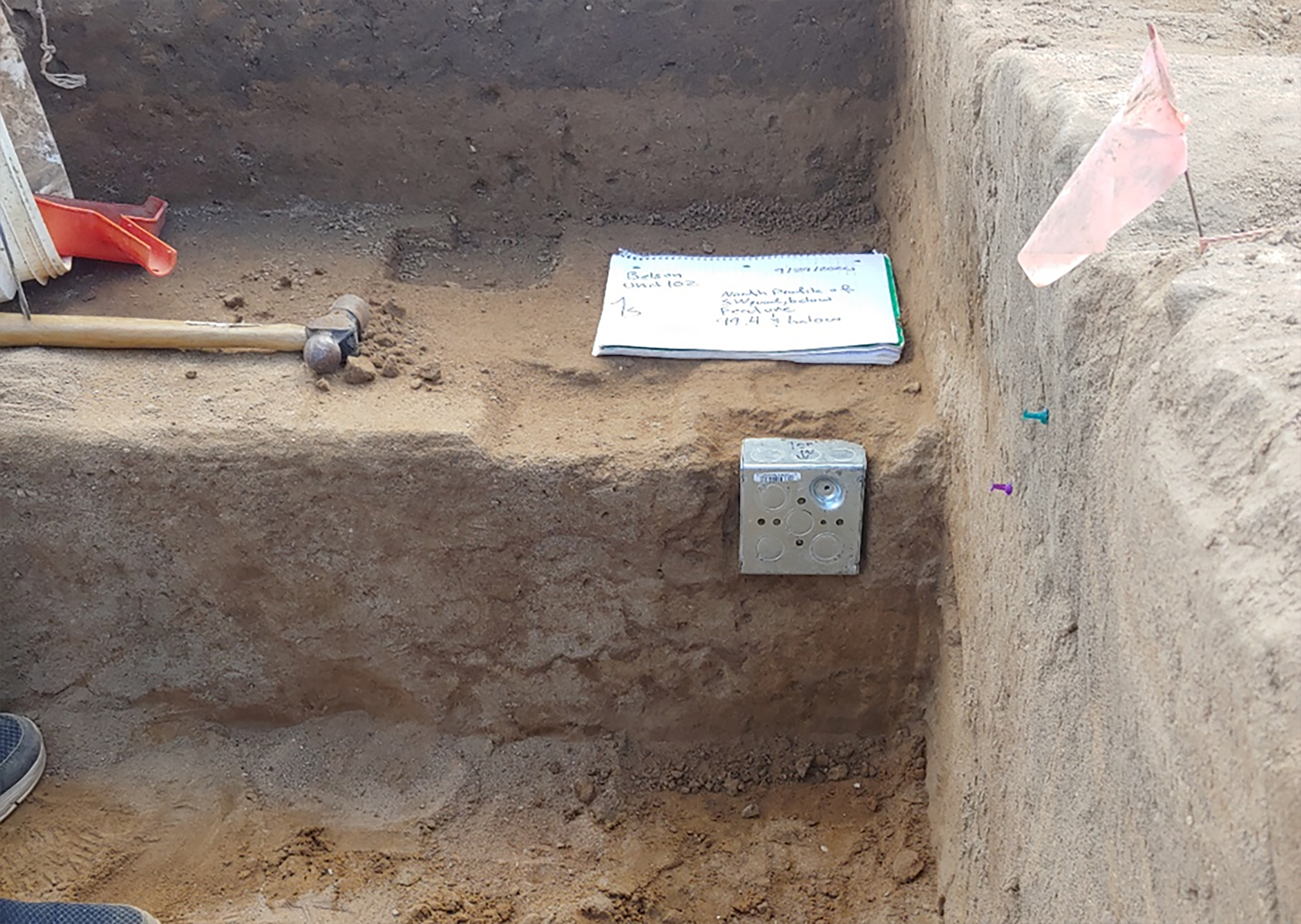

Supplement: S8 Fig — This image shows more accurate color of sediments and soil features associated with Feature 1, and a micromorphology sample in place before being removed. Note the difference between the soil texture and features after bisection of the unit. (TIF) [file pone.0302255.s008.tif]

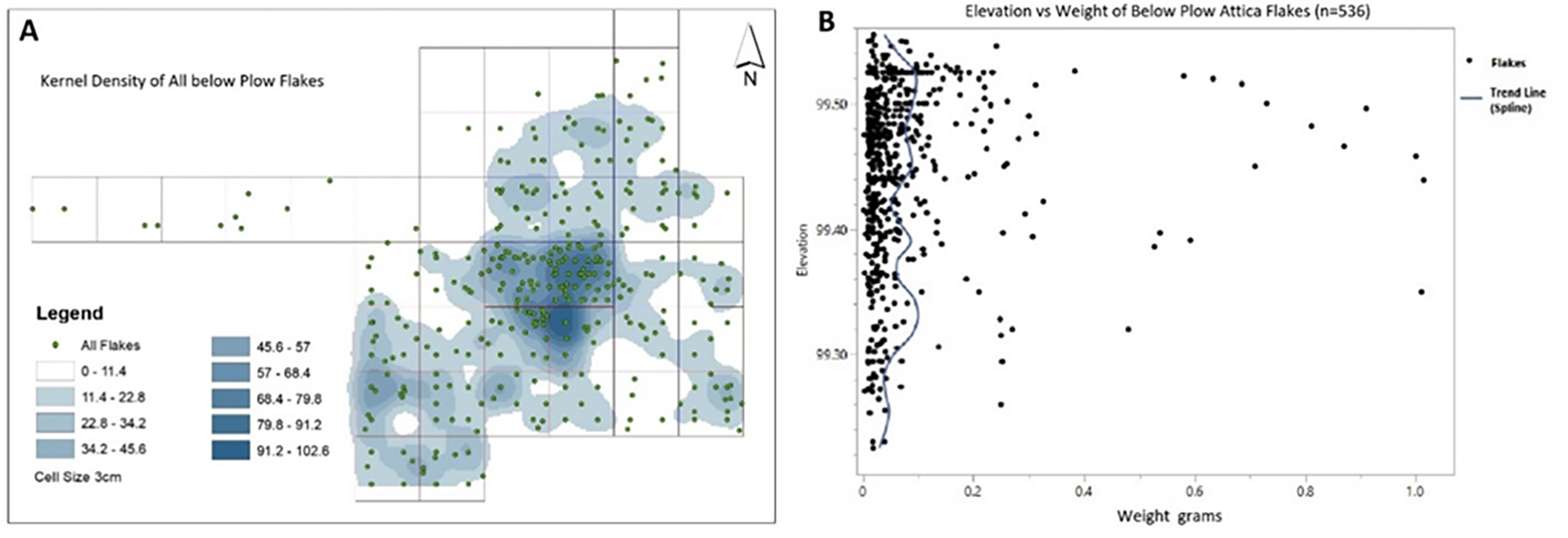

Supplement: S9 Fig — This diagram shows flake elevations with black dots and a blue trend line showing the relative changes in size (weight) with depth. It is important to note that the cultural deposit is not at a consistent elevation, and that a majority of the flakes below 99.35 are from the bottom portions of Features 1 and 2. (TIF) [file pone.0302255.s009.tif]

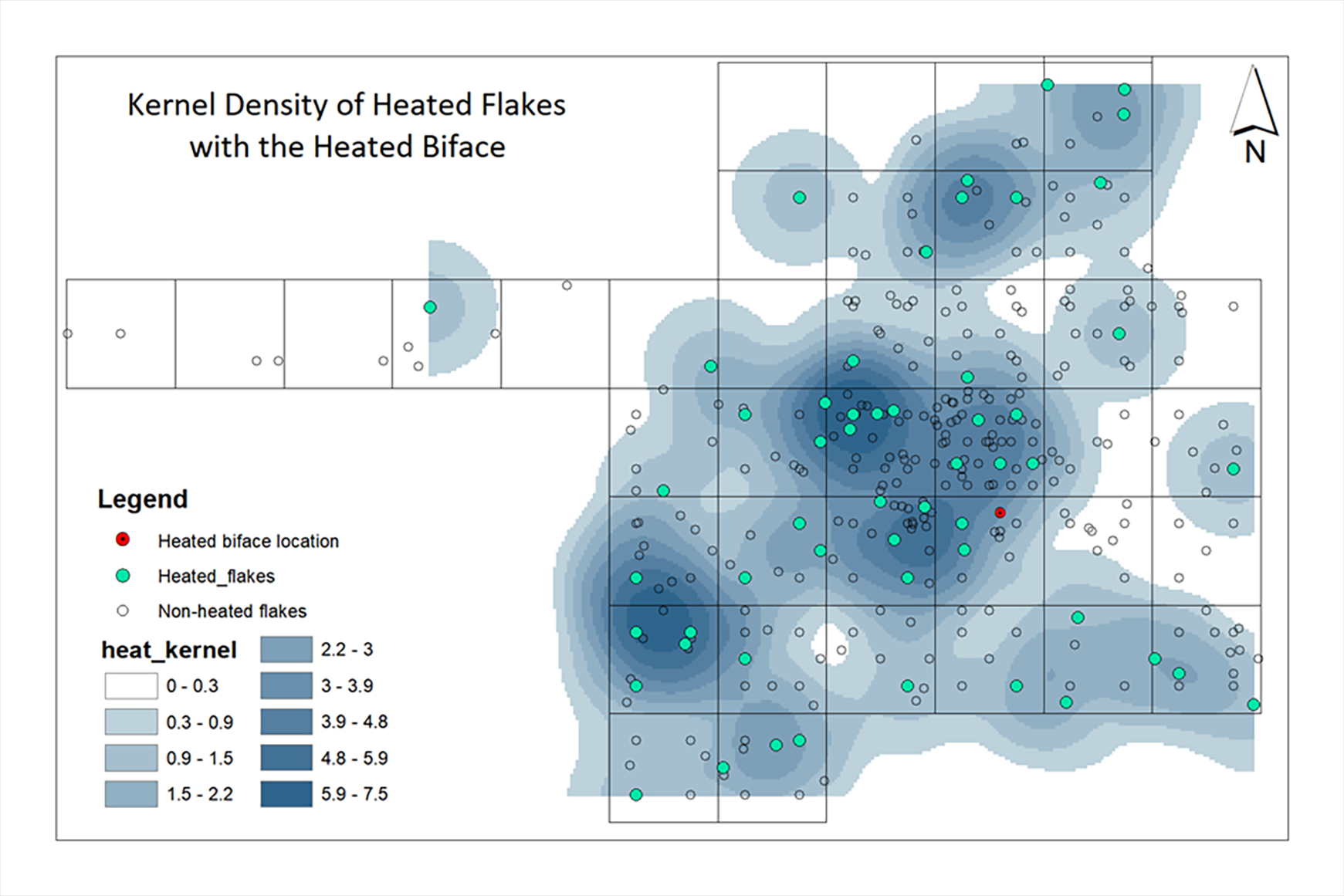

Supplement: S10 Fig — Map of heated flakes (teal dots), and non-heated flakes (open dots) over top of the kernel density map. Although it appears that the heated flakes concentrate in the feature areas, the percentage of flakes within and outside those areas is similar at just under 10%. Intended to show that there is an almost an even percentage of burnt flakes within Feature 1 as the rest of the excavation. (TIF) [file pone.0302255.s010.tif]

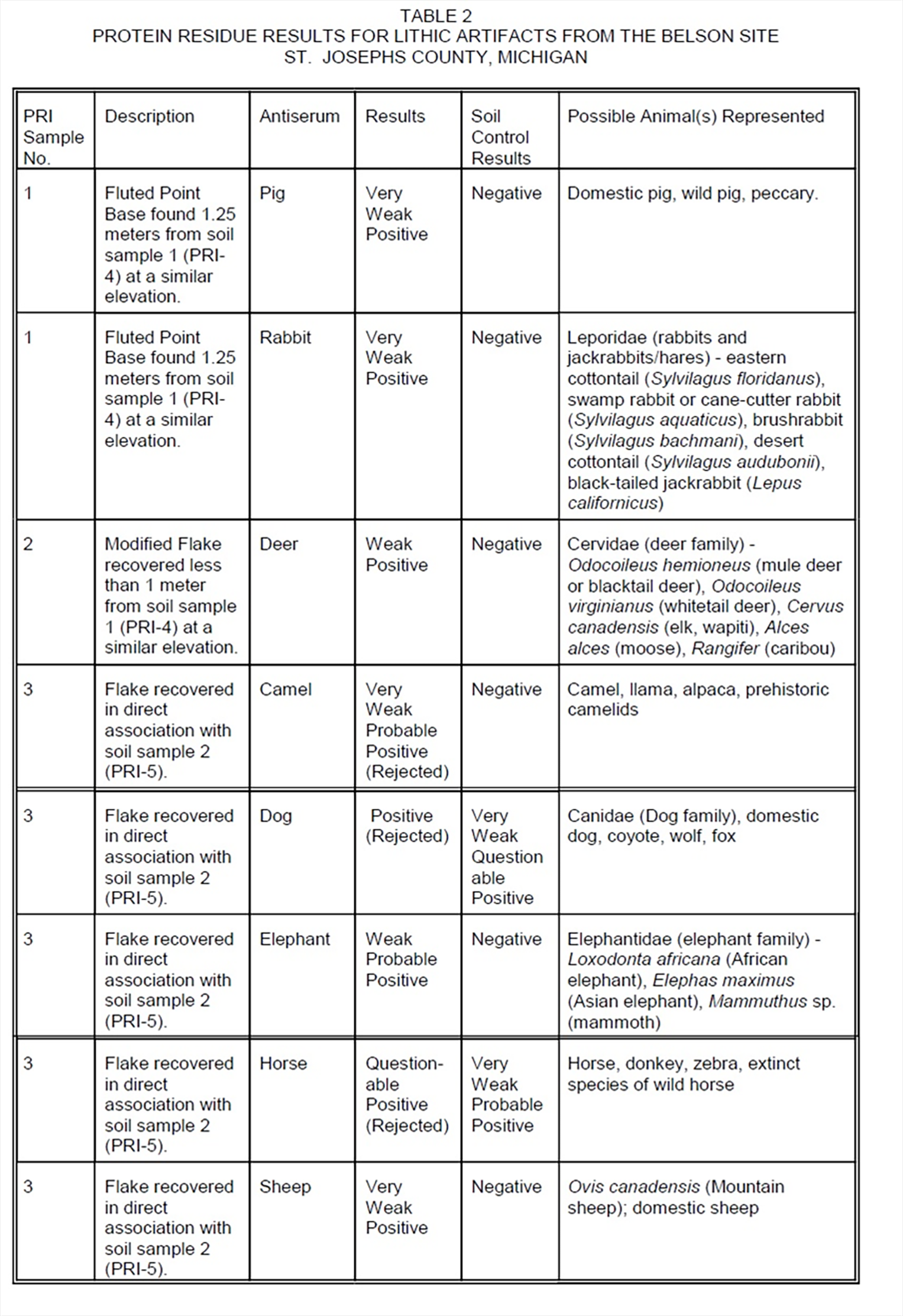

Supplement: S11 Fig — (TIF) [file pone.0302255.s011.tif]

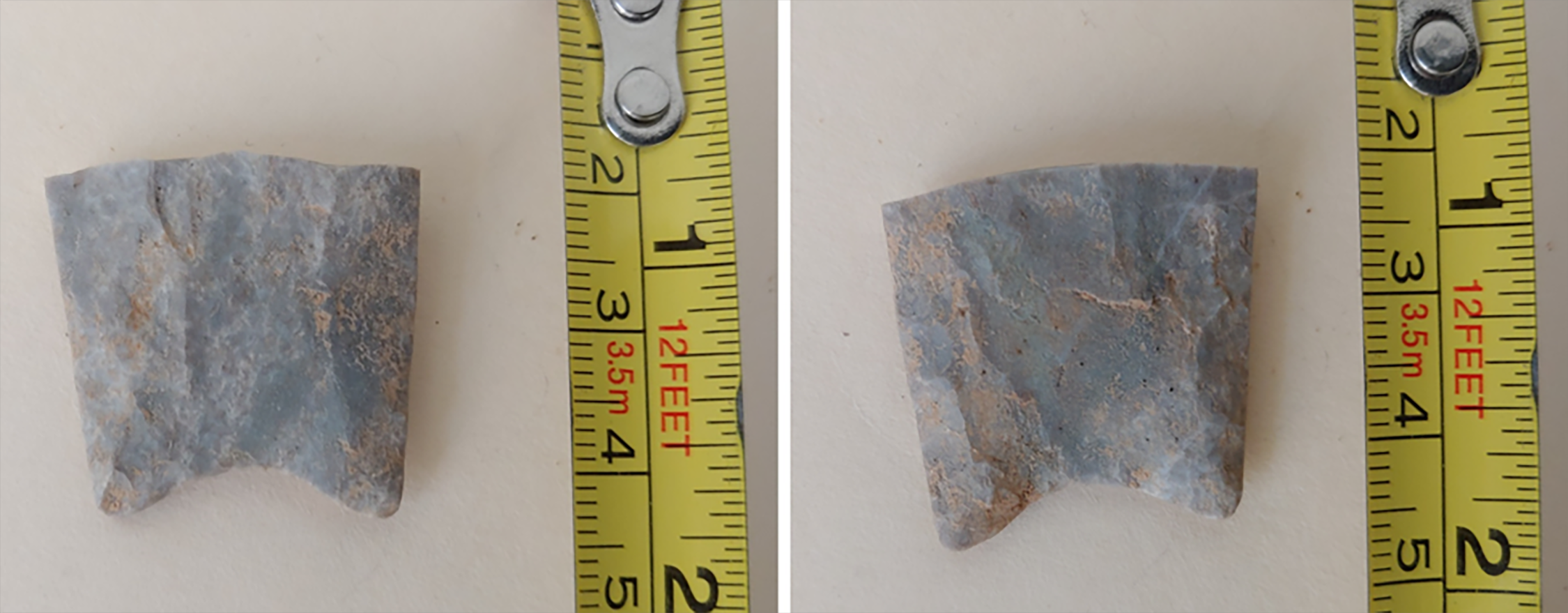

Supplement: S12 Fig — (TIF) [file pone.0302255.s012.tif]
