# Supplementary material for: Clovis organizational dynamics at a Late Glacial campsite in the central Great Lakes: Belson site excavations 2020–2021
Source: PLoS One. 2024 May 29;19(5):e0302255. doi: 10.1371/journal.pone.0302255 (PMC11135731; doi:10.1371/journal.pone.0302255)
Supplement: S2 Appendix — (DOCX) [file pone.0302255.s014.docx]

**S1 Appendix. Material and methods for the protein analysis from the PaleoResearch Lab.**

The lithics were washed using 0.5–1 ml of solution containing 0.02 M Tris hydrochloride, 0.5 M sodium chloride, and 0.5% Triton X-100 (Tris/NaCl/Triton). While in solution, the artifacts were placed in an ultrasonic bath for 30 minutes, on a rotating mixer for 30 minutes, back into an ultrasonic bath for an additional 30 minutes, and once again onto a rotating mixer for an additional 30 minutes. When removed from the ultrasonic bath, artifacts were rinsed using a small amount of reverse osmosis de-ionized (RODI) water to recover all of the protein wash solution.

Wash solutions were centrifuged to remove sediments and recover the solution, which was decanted into new microcentrifuge tubes. In addition, a soil control sample was tested because sediments contain compounds, such as bacteria and recent animal urine and/or feces, causing false positive results. These contaminants may originate in the sampled location or be introduced as air-borne material. One half gram of sediment associated with each artifact was added to 1 ml Tris/NaCl/Triton solution, and then refrigerated for several days prior to testing. The first step tests all residue washes extracted from the lithic artifacts and the sediment controls, when present, against pre-immune goat serum (serum from a non-immunized animal) to screen for the presence of non-specific, indiscriminate binding of proteins. All of the artifact washes tested negative against pre-immune serum. Next, the samples were tested against prepared animal and plant antisera obtained from a variety of commercial and private sources. Appropriate positive and negative controls were run for each antiserum. The blood of an animal for which the antiserum tests positively constitutes the positive control, while negative controls use the serum of the type of animal in which the antiserum was raised, either rabbit or goat.

Agarose gel poured onto GelBond® film acts as the medium for CIEP. Four columns of paired wells (2 mm in diameter separated by 3 mm of gel) organized in a series of eight rows were punched into the gel. The anodic (-) well contained the antiserum while the cathodic (+) well held the artifact’s protein extraction (the antigen). The sample was electrophoresed in Barbital buffer (pH 8.6) for 45 minutes at 130 V to drive the antigens and antibodies toward each other. Overnight, a 1 M NaCl bath removed extraneous proteins from the gel. The next morning the gel was pressed for 10 minutes, rinsed with RODI water for an hour, and then pressed for an additional 10 minutes to remove extraneous water and provide a rinse to remove the NaCl. The gels were air dried.

A positive reaction appears as a vertical line of precipitation between the two wells. Coomassie Blue stain was used to make the line of precipitation easier to see. When a positive reaction was obtained between the artifact wash (antigen) and an antiserum at the 1:5 dilution, the antigen from the artifact was retested and the soil control was tested using dilute antiserum at a concentration of 1:5. Retests distinguish between true and false positives, identifying a true positive when they replicate the initial positive reaction and when that reaction is not observed in the accompanying soil control sample. Positive reactions obtained after the second test with dilute antisera were reported.

Many archaeological samples do not produce the expected clear vertical lines of precipitation that are observed with positive blood-based controls. Therefore, descriptions, based on the presence and pattern of precipitation lines, and reaction strengths for each dilution level were recorded to help monitor consistency and viability of the reactions between antisera and archaeological proteins. A recorded “positive” result displays a clear vertical precipitation line between the antiserum and the sample (antigen), indicating the sample wash contained proteins related to the animal represented by the antiserum, or a member of its family group/order. A “weak positive” demonstrates a faint precipitation line. This suggests presence of deteriorated proteins similar to the antiserum animal’s family or order. “Probable positive” samples produce a fuzzy precipitation line or curved concentrated cloud of stain during testing. These reactions suggest the presence of degraded proteins related to the animal represented by the antiserum. However, this reaction cannot be assigned as a definitive positive. Reactions lacking vertical precipitation lines, such as a dense cloud of stain concentrated between the anodic and cathodic wells, are recorded as “questionable positives.”
