# Supplementary material for: Clovis organizational dynamics at a Late Glacial campsite in the central Great Lakes: Belson site excavations 2020–2021
Source: PLoS One. 2024 May 29;19(5):e0302255. doi: 10.1371/journal.pone.0302255 (PMC11135731; doi:10.1371/journal.pone.0302255)
Supplement: S1 Text — (DOCX) [file pone.0302255.s016.docx]

Supplemental Information

**S1 Text. Introduction to supplementary information.**

The supplemental material section will consist of four parts. Frist, additional photographs of the site and excavation will be presented to further show the site and excavation context (S1 Fig – S4 Fig). Second, additional photographs of Feature 1 will be provided to better show how it appeared in the field (S5 Fig – S8 Fig). Third, additional information about the sub-plow deposit, including potential limited size sorting, and the occurrence of heated flakes (S9 Fig – S10 Fig). Fourth, is a summary of protein analysis laboratory procedures and the table of results from protein analysis provided by PaleoResearch (Scott Cummings and Maison 2021) (S1 Appendix, and S11 Fig). Fifth is an image of one of the two fluted bifaces excavated from Belson (S12 Fig). Sixth, the Chi Square tests performed in Excell 365 to test the significant of the pattern of flake distribution of white/green flakes being deeper on average than the blue/gray and other flake types (S13 Fig). Finally, all the sub-plow flake data used in the analysis will be presented (S1 Dataset).

**S1 Fig.** **Drone photograph from season 1 in 2020 looking northwest**. Intended to present the excavation as it appeared in the field near the end of the excavation seasons, and to give readers a visual understanding of the site and excavation context. (Photo credit Tommy Talbot)

**S2 Fig.** **Drone photograph from season 1 in 2020 looking down.** The photograph is oriented south up. Intended to present the excavation as it appeared in the field near the end of the excavation seasons, and to give readers a better visual understanding of the site and excavation context. (Photo credit Tommy Talbot)

**S3 Fig. Belson central cluster excavation in September 2020**. The initial N-S and E-W trenches are filled (north up). Feature 1 is cut by shadowed black square southwest of their juncture (Photo Credit Tommy Talbot).

**S4 Fig.** **Drone photograph from season 2 in 2021 looking down.** The photograph is oriented with North to the right. Intended to present the excavation as it appeared in the field, and to give readers a visual understanding of the site and excavation context. (Photo credit Tommy Talbot)

**S5 Fig.** **Unit 102 before bisection of Feature 1.** Show unit 102 after removal of the plow zone and first 10 cm of overlying sediment, but before bisection. Also, Dr. Wrights legs and scarf.

**S6 Fig.** **Image of the north face of Unit 102.** Shows the north face of unit 102 before bisection to show Feature 1 in profile. Note that the profile is mostly loam without other pedogenic features. This will change after bisection.

**S7 Fig.** **Image of the north face of Unit 102 after bisection.** This image shows unit 102 after bisection in the east-west direction to show Feature 1 in profile. Note the pedogenic features including clay patches, and iron oxide accumulations.

**S8 Fig.** **Additional photograph of unit 102 bisected to show Feature 1 in profile**. This image shows more accurate color of sediments and soil features associated with Feature 1, and a micromorphology sample in place before being removed. Note the difference between the soil texture and features after bisection of the unit.

**S9 Fig.** **Size sorting diagram.** This diagram shows flake elevations with black dots and a blue trend line showing the relative changes in size (weight) with depth. It is important to note that the cultural deposit is not at a consistent elevation, and that a majority of the flakes below 99.35 are from the bottom portions of Features 1 and 2.

**S10 Fig.** **Kernel density map of heated flakes.** Map of heated flakes (teal dots), and non-heated flakes (open dots) over top of the kernel density map. Although it appears that the heated flakes concentrate in the feature areas, the percentage of flakes within and outside those areas is similar at just under 10%. Intended to show that there is an almost an even percentage of burnt flakes within Feature 1 as the rest of the excavation.

**S11 Fig**. **Summary of results table from the Paleo Research lab in Colorado.**

**S12 Fig. Image of both sides of the excavated Attica fluted point base 7E.**

**S13 Fig. Chi Square Statistics performed in Excell 365 using the CHISQ.TEST function for Features 1, 2 and the excavation block as a whole.**
